# miRNA863-3p sequentially targets negative immune regulator *ARLPKs* and positive regulator *SERRATE* upon bacterial infection

**DOI:** 10.1038/ncomms11324

**Published:** 2016-04-25

**Authors:** Dongdong Niu, Yifan E. Lii, Padmanabhan Chellappan, Lei Lei, Karl Peralta, Chunhao Jiang, Jianhua Guo, Gitta Coaker, Hailing Jin

**Affiliations:** 1 Department of Plant Protection, Nanjing Agriculture University, Nanjing 210095, China; 2 Department of Plant Pathology and Microbiology, Center for Plant Cell Biology and Institute for Integrative Genome Biology, University of California, Riverside, California 92521, USA; 3 Department of Plant Pathology, University of California, Davis, California 95616, USA

## Abstract

Plant small RNAs play important roles in gene regulation during pathogen infection. Here we show that miR863-3p is induced by the bacterial pathogen *Pseudomonas syringae* carrying various effectors. Early during infection, miR863-3p silences two negative regulators of plant defence, atypical receptor-like pseudokinase1 (*ARLPK1*) and *ARLPK2*, both lacking extracellular domains and kinase activity, through mRNA degradation to promote immunity. ARLPK1 associates with, and may function through another negative immune regulator ARLPK1-interacting receptor-like kinase 1 (AKIK1), an active kinase with an extracellular domain. Later during infection, miR863-3p silences SERRATE, which is essential for miRNA accumulation and positively regulates defence, through translational inhibition. This results in decreased miR863-3p levels, thus forming a negative feedback loop to attenuate immune responses after successful defence. This is an example of a miRNA that sequentially targets both negative and positive regulators of immunity through two modes of action to fine-tune the timing and amplitude of defence responses.

## Abstract (web summary)

Small RNA plays an important role in regulating the plant defence against bacterial pathogens. Here the authors propose that miR863-3p acts to fine-tune the timing of defence responses by sequentially silencing negative and positive regulators of the plant immune response.

## Graphical abstract

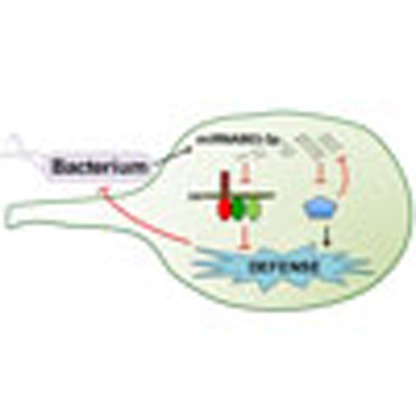

During pathogen infection, plants undergo reprogramming and fine-tuning of gene expression to activate innate immune responses, and small RNAs (sRNAs) are important regulators in this process^1^,^2^,^3^,^4^. As the first layer of defence, plant pattern-recognition receptors detect conserved pathogen features called pathogen-associated molecular patterns (PAMPs), leading to PAMP-triggered immunity (PTI)^5^. Some pathogens deliver effector proteins to attenuate PTI; however, in resistant hosts, Nod-like receptor (NLR) proteins recognize cognate effectors and activate effector-triggered immunity (ETI), which is more specific, robust and rapid than PTI. ETI is often characterized by the hypersensitive response (HR), a type of cell death, at the site of infection^6^,^7^. One well-studied effector-NLR model in *Arabidopsis* is the effector AvrRpt2 from the bacterial pathogen *Pseudomonas syringae* pv. *tomato* (*Pst*) DC3000 and its cognate plant host NLR protein RPS2 (refs 8, 9).

sRNAs are critical regulators of plant defence responses and pathogen virulence^1^,^2^,^3^,^4^,^10^. These microRNAs (miRNAs) and short interfering RNAs (siRNAs) are processed by Dicer or Dicer-like (DCL) proteins and then incorporated into Argonaute (AGO) proteins to form RNA-induced silencing complex to target genes with full or partial complementary sequences. sRNAs can induce gene silencing by guiding post-transcriptional gene silencing via mRNA degradation or translational inhibition, or transcriptional inhibition via DNA methylation or chromatin modification^11^. During infection with *Pst* DC3000 carrying the effector gene *avrRpt2*, the natural antisense transcript-associated siRNA natsiRNAATGB2 silences a gene with pentatricopeptide repeats, which acts as a negative regulator of ETI mediated by *RPS2* (ref. 12). A long siRNA, AtlsiRNA, also induced by *Pst* (*avrRpt2*), silences the *AtRAP* gene (*Arabidopsis thaliana RNA-*binding domain abundant in apicomplexans), which has a negative role in defence^13^. miR393 contributes to PTI by targeting auxin signalling receptors upon elicitor flg22 treatment or after infection with virulent *Pst* DC3000 (ref. 14). The complementary strand of miR393 within the sRNA duplex, miR393*, regulates ETI during infection of *Pst* (*avrRpt2*) by targeting a protein trafficking gene *MEMB12* (Membrin 12) to promote the secretion of antimicrobial PR (pathogenesis-related) proteins^15^. miR160a enhances PAMP-induced callose deposition by targeting auxin response factors^16^. In addition to these examples of sRNAs that are induced by pathogen infection, some sRNAs are downregulated by infection. For example, miR398b, which targets a cytochrome c oxidase and two copper superoxide dismutases, and miR773, which targets a DNA methyltransferase, *MET2*, both negatively regulate PAMP-induced callose deposition, as well as PTI against *Pst* (EV) and *Pst* (Δ*hrcC*)^16^. Collectively, these studies highlight the importance of sRNAs that target critical plant genes involved in immune signalling. However, no sRNAs are reported to target genes with antagonistic roles in plant immunity or any other cellular processes.

We previously identified miRNAs and siRNAs that are differentially expressed on infection with various bacterial *Pst* strains^17^. One of these miRNAs, miR863-3p, was highly upregulated by the avirulent strain *Pst* (*avrRpt2*); thus, we hypothesized that this miRNA may play an important role in ETI, and sought to characterize its targets and their function. Here we show that miR863-3p targets the putative pseudokinases, atypical receptor-like pseudokinase 1 (*ARLPK1*) and *ARLPK2*, early on during infection to boost plant defence. ARLPK1 interacts with ARLPK1-interacting receptor-like kinase (AKIK1), an active kinase that also negatively regulates defence. Later during infection, miR863-3p silences SERRATE (SE)—a protein important for miRNA processing and accumulation^18^,^19^,^20^,^21^ and for defence—to form a negative feedback loop to regulate the level of miRNA863-3p. Attenuation of immune responses after successful defence is possibly to conserve host resources for growth and reproduction.

## Results

### miR863-3p is induced by infection with avirulent *Pst* strains

**Figure 1:**
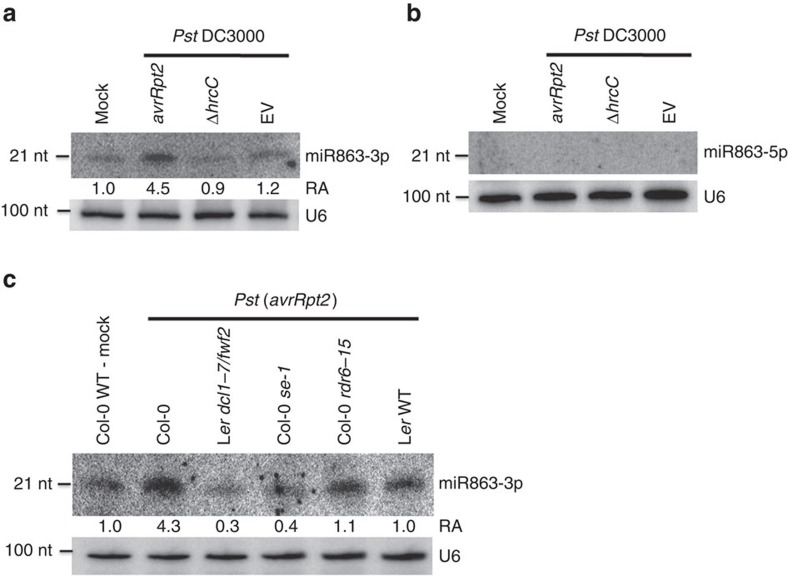
miR863-3p is induced by *Pst* carrying the effector avrRpt2. (**a**) miR863-3p and (**b**) miR863-5p levels in mock-, *Pst* (*avrRpt2*)-, *Pst* (Δ*hrcC*)- and *Pst* (EV)-infected Col-0 WT plants were detected by northern blot analysis. Bacterial inoculum concentration: 1 × 10^7^ c.f.u. per ml. Leaf tissue was collected at 14 h.p.i. U6 was used as a loading control. Relative abundance (RA) levels are indicated. Similar results were obtained from three biological replicates. (**c**) miR863-3p levels in various *Pst* (*avrRpt2*)-infected mutants defective in miRNA biogenesis (L*er dcl1-7/fwf2* and Col-0 *se-1*) or siRNA biogenesis (Col-0 *rdr6-15*) are detected by northern blot analysis. Mock-treated and *Pst* (*avrRpt2*)-infected Col-0 WT and *Pst* (*avrRpt2*)-infected L*er* WT were used as controls. Bacterial inoculum concentration: 1 × 10^7^ c.f.u. per ml. Leaf tissue was collected at 14 h.p.i. U6 was used as a loading control. RA levels are indicated. Similar results were obtained from three biological replicates.

To identify miRNAs involved in regulating immune responses to bacterial infection, we previously profiled sRNAs from plants challenged with virulent and avirulent bacterial strains, *Pst* (EV) and *Pst* (*avrRpt2*), respectively^17^. We found that miR863-3p was highly upregulated during *Pst* (*avrRpt2*) infection. miR863-3p is a 21-nucleotide (nt) miRNA originating from the 3′-arm of the precursor miRNA (Supplementary Fig. 1a)^22^. Northern blot analysis confirmed that miR863-3p was induced by an avirulent strain *Pst* (*avrRpt2*), but not by the virulent strain *Pst* carrying an empty vector (EV), a strain that has a mutation in its type III secretion system—*Pst* (Δ*hrcC*), or mock solution (10 mM MgCl_2_; Fig. 1a). miR863-3p levels are also upregulated by avirulent strains *Pst* (*avrRpm1*) and *Pst* (*avrRps4*), although to a lesser degree compared with *Pst* (*avrRpt2*) (Supplementary Fig. 1b). Interestingly, miR863-5p, the complementary strand of miR863-3p, was not induced by any of these *Pst* strains, and thus is not likely to be involved in defence against *Pst* (Fig. 1b).

Biogenesis of miRNAs depends on DCL1, which processes pri-miRNAs, and is aided by interacting RNA-binding proteins HYL1 and SE to promote accurate processing^20^. Northern blot analysis revealed that miR863-3p accumulation is dependent on DCL1 and SE, but independent of RDR6, which is involved in the biogenesis and amplification of siRNAs (Fig. 1c)^11^. These results indicate that miR863-3p is indeed a true miRNA instead of a siRNA. Taken together, our results show that miR863-3p is dependent on DCL1 and SE, and it is induced by challenge with avirulent *Pst* strains, especially *Pst* (*avrRpt2*). Its complementary strand was not detected in our experiments, showing that miR863-3p is the stable miRNA strand during pathogen infection.

### miR863-3p targets two ARLPKs and SE

**Figure 2:**
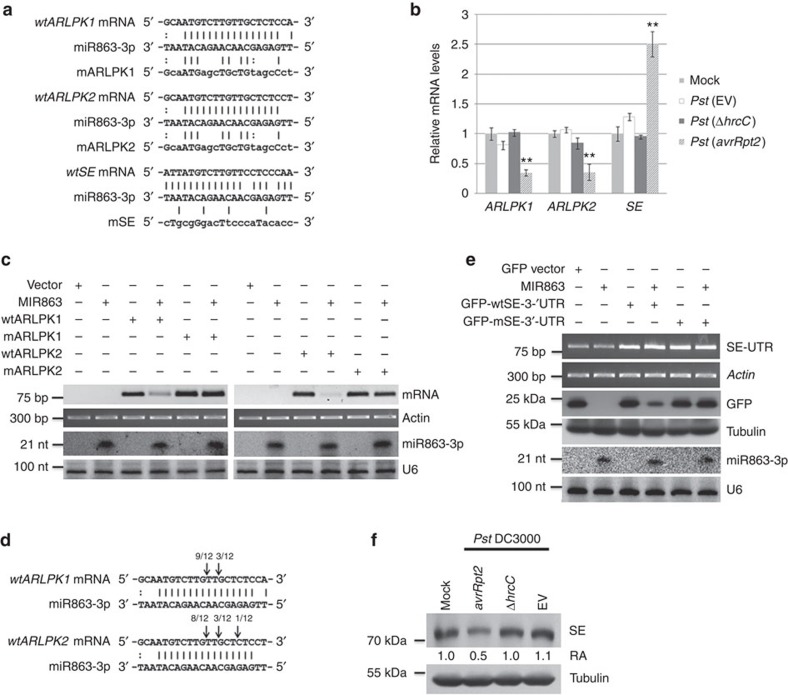
miR863-3p targets and silences *ARLPK1* and *ARLPK2* and SE by two modes of action. (**a**) Nucleotide sequences of wt and mutated (m) versions of *ARLPK1*, *ARLPK2* and *SE* aligned against the miR863-3p sequence. (**b**) Relative expression levels of *ARLPK1*, *ARLPK2* and *SE* transcripts in mock- and *Pst* (EV), *Pst* (Δ*hrcC*) and *Pst* (*avrRpt2*)-infected Col-0 WT plants were detected by real-time RT–PCR. Bacterial inoculum concentration: 1 × 10^7^ c.f.u. per ml. Leaf tissue was collected 14 h.p.i. Relative mRNA levels in mock-infected plants were set at 1. Error bars indicate s.d. from three technical replicates. Similar results were obtained from three biological replicates. **P value <0.01 (Student's *t*-test). (**c**) *Agrobacterium*-mediated transient co-expression assay with WT (wtARLPK1 and wtARLPK2) or mutated kinases (mARLPK1 and mARLPK2) and MIR863. mRNA levels of the wt and mutant kinases were detected by RT–PCR. rRNA was used as a loading control. miR863-3p levels were detected by northern blot analysis. U6 was used as a loading control. Similar results were obtained from two biological replicates. (**d**) Cleavage sites of *ARLPK1* and *ARLPK2* mRNAs were revealed by mapping cloned RLM-5′-RACE products using *Pst* (*avrRpt2*)-infected WT *Arabidopsis* plants. Arrows indicate positions and proportions of clones mapping to the sites. (**e**) *Agrobacterium*-mediated transient co-expression assay with GFP-tagged wt (GFP-wtSE-3′-UTR) or mutated (GFP-mSE-3′-UTR) 3′-UTR SE fragment and MIR863. wtSE-GFP and mSE-GFP mRNA were detected by RT–PCR using primers specific to the SE 3′-UTR. Actin was used as a loading control. GFP protein levels were detected by western blot. α-Tubulin was used as a loading control. miR863-3p levels were detected by northern blot analysis. U6 was used as a loading control. Similar results were obtained from two biological replicates. (**f**) SE protein levels in mock-, *Pst* (*avrRpt2*)-, *Pst* (Δ*hrcC*)- and *Pst* (EV)-infected Col-0 WT plants were detected by western blot using an anti-SE antibody. Bacterial inoculum concentration: 1 × 10^7^ c.f.u. per ml. Leaf tissue was collected at 14 h.p.i. α-Tubulin was used as a loading control. Relative abundance (RA) levels are indicated. Similar results were obtained from two biological replicates.

We hypothesized that miR863-3p targets genes involved in immune responses, but since miR863-3p has no validated targets in existing databases, we predicted its potential targets (Methods). miR863-3p potentially targets the coding sequences of two atypical receptor-like kinases (RLKs), *ARLPK1* and *ARLPK2*, belonging to the leucine-rich repeat (LRR) III RLK subfamily^23^, as well as the 3′-untranslated region (UTR) of *SE*, which encodes a zinc-finger protein found in both the miRNA biogenesis DCL1 complex^19^, and the nuclear cap-binding protein complex^24^ (Fig. 2a). Typical RLKs have an extracellular ligand-binding domain, a single transmembrane region and an intracellular kinase domain^23^. Both *ARLPK1* and *ARLPK2* have a signal peptide sequence overlapping with their N-terminal transmembrane domain (Supplementary Fig. 2a,b) and lack extracellular domains^23^. They also have variations in the conserved features of functional kinase domains such as the glycine-rich loop, VAIK motif and HRD motif (Supplementary Fig. 3), and are thus predicted pseudokinases^25^. Because ARLPK1 and ARLPK2 are missing conserved key residues in the consensus motifs and are lacking extracellular domains, we named them atypical receptor-like pseudokinases (*ARLPK1*, At5g61570; and *ARLPK2* At5g07620). miR863-3p is also predicted to target a third gene *SE*, which is involved in the accumulation of miRNAs, including miR863-3p (Fig. 1c). During *Pst* (*avrRpt2*) infection, the mRNA levels of the two *ARLPKs* are downregulated compared with mock-infected plants; however, *SE* mRNA levels are upregulated. In contrast, the levels of all three transcripts do not change significantly during *Pst* (EV) or *Pst* (Δ*hrcC*) infection (Fig. 2b).

To confirm that *ARLPK1* and *ARLPK2* are true targets of miR863-3p, we performed *Agrobacterium*-mediated transient co-expression assays in *Nicotiana benthamiana* using wild-type (wt) and mutated (m) kinase genes. The mutated kinases have an altered miR863-3p target site, yet the predicted amino-acid sequence is unchanged (Fig. 2a). MIR863 co-expression with wtARLPK1 or wtARLPK2 resulted in a decrease of the mRNA levels of both kinases, while co-expression with mARLPK1 or mARLPK2 did not affect the mRNA levels of *mARLPK1* or *mARLPK2* (Fig. 2c). In addition, we performed RNA-ligase mediated 5' rapid amplification of cDNA ends (RLM-RACE) in *Pst* (*avrRpt2*)-infected *Arabidopsis* to map miR863-3p-guided cleavage sites in *ARLPK* mRNA. As expected, the majority of the 5′-RACE cloned products mapped to the predicted target site in *ARLPK1* and *ARLPK2* mRNAs (Fig. 2d). These data indicate *ARLPK1* and *ARLPK2* are direct targets of miR863-3p, and they are silenced via mRNA cleavage and degradation.

In plants, most miRNAs silence their targets by directing mRNA cleavage and degradation, while others mediate translational inhibition^26^. In mammals, most miRNA target sites for translational inhibition are located in the 3′-UTR, and the target site of miR863-3p in *SE* is also at the beginning of the 3′-UTR. *SE* mRNA levels are not downregulated by miR863-3p (Fig. 2b), even though the nucleotide alignment shows very few mismatches (Fig. 2a). Thus, it is possible that miR863-3p silences SE through translational inhibition. In mammals, target mRNAs silenced by RNA-induced silencing complex via translational repression are delivered to and accumulate in processing (P)-bodies^27^. Thus, it is possible that we see elevated *SE* transcripts not only because of continual transcription of *SE* due to the absence of SE protein product but also because of the accumulation of sequestered *SE* mRNAs in P-bodies. To test that hypothesis, we generated constructs of green fluorescent protein (GFP) fused to a wt version of truncated *SE* that includes only the 3′-UTR (GFP-wtSE-3′-UTR) or a mutated truncated version of *SE* with an altered miRNA target site (GFP-mSE-3′-UTR; Fig. 2a). We co-expressed the fusions with MIR863 in *N. benthamiana*, and western blotting revealed that GFP-wtSE-3′-UTR, but not GFP-mSE-3′-UTR, levels were downregulated (Fig. 2e). To confirm this result *in vivo*, we obtained an anti-SE antibody and detected SE protein levels in *Arabidopsis* plants challenged with *Pst* strains. Western blot analysis revealed that SE protein levels are downregulated by *Pst* (*avrRpt2*) but not by *Pst* (Δ*hrcC*) or *Pst* (EV) (Fig. 2f). These results suggest that miR863-3p targets and silences SE by inhibiting its translation. Thus, miR863-3p uses two different modes of action to silence its targets—it suppresses the two *ARLPKs* via mRNA cleavage and degradation and inhibits *SE* by translational inhibition.

### ARLPKs are negative regulators of disease resistance

**Figure 3:**
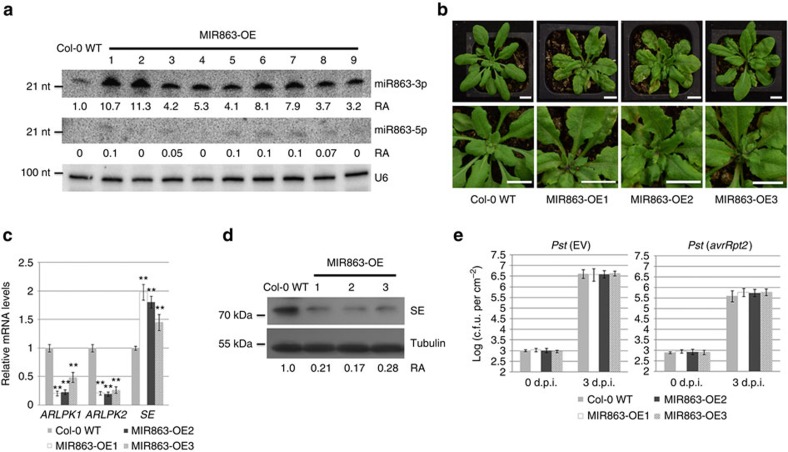
Plants overexpressing MIR863 show serrate leaves and have downregulated *ARLPK1* and *ARLPK2* mRNA and SE protein levels. (**a**) miRNA863-3p and miRNA863-5p levels were detected in Col-0 WT and MIR863-OE lines #1–9. U6 was used as a loading control. Relative abundance (RA) levels are indicated. miRNA863-5p RA levels were measured compared with the miR863-3p levels in Col-0 WT. Similar results were obtained from two biological replicates. (**b**) Phenotypes of 4-week-old MIR863 OE plants. Out of the 20 lines tested, 4 highly expressed lines resembled the *serrate* mutant phenotype (MIR863-OE1 and -OE2 are shown), while 7 medium expressed lines showed a weak serrated leaf phenotype (MIR863-OE3 is shown). Scale bar, 10 mm. (**c**) *ARLPK1*, *ARLPK2* and *SE* transcript levels in Col-0 WT and MIR863-OE plants were detected by real-time RT–PCR. Error bars indicate s.d. from three technical replicates. Similar results were obtained from three biological replicates. **P value <0.01 (Student's *t*-test). (**d**) SE protein levels were detected in *Pst* (*avrRpt2*)-infected Col-0 WT and MIR863-OE plants by western blot analysis using an anti-SE antibody. Bacterial inoculum concentration: 1 × 10^7^ c.f.u. per ml. Leaf tissue was collected at 14 h.p.i. α-Tubulin was used as a loading control. RA levels are indicated. Similar results were obtained from two biological replicates. (**e**) Bacterial growth in *Pst* (EV)- and *Pst* (*avrRpt2*)-infected Col-0 WT and MIR863-OE plants. Bacterial inoculum concentration: 5 × 10^5^ c.f.u. per ml. Bacterial growth was measured at 3 d.p.i. Error bars represent s.d. for at least 15 leaf discs. Similar results were obtained from three biological replicates.

To investigate the role of miR863-3p during plant immune responses, we created transgenic plants overexpressing MIR863 under the control of the cauliflower mosaic virus 35S promoter. Figure 3a shows nine such overexpression (OE) lines. The lines with very high miR863-3p expression (such as MIR863-OE1 and -OE2 lines) resembled the *serrate* mutant phenotype^28^, further supporting that SE is indeed a miR863-3p target (Fig. 3b). Only a slight increase of miR863-3p was observed after infection with *Pst* (*avrRpt2*) in the OE lines, most likely because miR863-3p levels are already very high (Supplementary Fig. 4). We also detected very low levels of miR863-5p in some of the OE lines (Fig. 3a). We chose two transgenic lines with high miR863-3p levels, MIR863-OE1 and -OE2, and one with lower levels, MIR863-OE3, for functional analyses (Fig. 3a). MIR863-OE1 and -OE2 plants were slightly smaller than Col-0 WT plants (Fig. 3b, upper panels). In the MIR863 overexpression lines, *ARLPK1* and *ARLPK2* mRNA levels are downregulated while *SE* mRNA levels are elevated, as shown by real-time reverse transcription–PCR (RT–PCR) results (Fig. 3c). *SE* mRNA levels are elevated just as in *Pst* (*avrRpt2*)-infected plants (Fig. 2a), most likely due to feedback compensation to increase transcription and also sequestering of transcripts in P-bodies^27^. SE protein levels in the overexpression lines are significantly lower compared with Col-0 WT, especially in MIR863-OE1 and -OE2 (Fig. 3d). Interestingly, even though the target genes were downregulated, the MIR863 overexpression lines showed no altered disease resistance to *Pst* (EV) or *Pst* (*avrRpt2*) compared with Col-0 WT plants (Fig. 3e).

**Figure 4:**
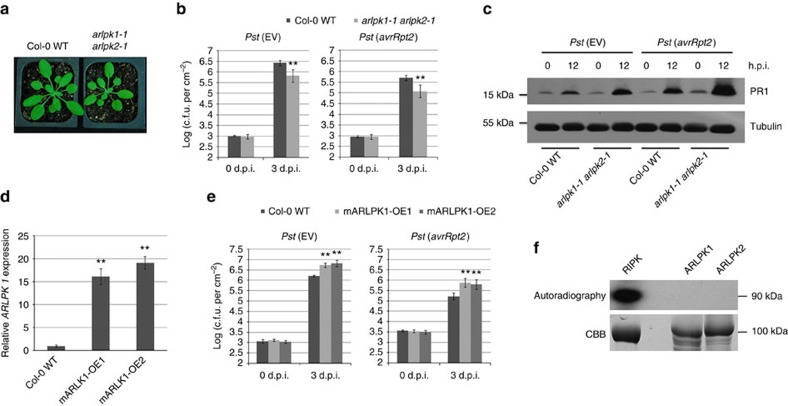
Analysis of the *arlpk1-1 arlpk2-1* double mutant. (**a**) Phenotype of 4-week-old *arlpk1-1 arlpk2-1* double mutants compared with Col-0 WT plants. (**b**) Bacterial growth in *Pst* (EV)- and *Pst* (*avrRpt2*)-infected Col-0 WT and *arlpk1-1 arlpk2-1* double mutants. Bacterial inoculum concentration: 5 × 10^5^ c.f.u. per ml. Bacterial growth was measured at 3 d.p.i. Error bars represent s.d. for at least 15 leaf discs. Similar results were obtained in three biological replicates. **P value <0.01 (Student's *t*-test). (**c**) PR1 protein levels were detected in *Pst* (EV)- and *Pst* (*avrRpt2*)-infected Col-0 WT and *arlpk1-1 arlpk2-1* double mutants by western blot using an anti-PR1 antibody. Bacterial inoculum concentration: 1 × 10^7^ c.f.u. per ml. Samples were collected at 0 and 12 h.p.i. α-Tubulin was used as a loading control. Similar results were obtained from two biological replicates. (**d**) Levels of *ARLPK1* transcript in Col-0 WT, mARLPK1-OE1 and mARLPK1-OE2 lines were detected by real-time RT–PCR. Error bars indicate s.d. from three technical replicates. Similar results were obtained in three biological replicates. **P value <0.01 (Student's *t*-test). (**e**) Bacterial growth in *Pst* (EV)- and *Pst* (*avrRpt2*)-infected Col-0 WT and mARLPK1-OE plants. Bacterial inoculum concentration: 5 × 10^5^ c.f.u. per ml. Bacterial growth was measured 3 d.p.i. Error bars represent s.d. for at least 15 leaf discs. Similar results were obtained from three biological replicates. **P value <0.01 (Student's *t*-test). (**f**) Recombinant MBP-tagged kinase domains of RIPK, ARLPK1 and ARLPK2 proteins were subjected to a radioactive kinase assay and kinase activity was detected using autoradiography. Bottom panel: SDS–PAGE gel stained with coomassie brilliant blue (CBB) demonstrating protein abundance. Similar results were obtained from two biological replicates.

To understand why overexpressing MIR863 did not have an effect on the defence response, we decided to study the function of miR863-3p targets individually. First, we focused on the functional analysis of the two ARLPKs and obtained the null mutants SALK_144635 (*arlpk1-1*) and SALK_040744C (*arlpk2-1*)^29^. Each mutant contains a T-DNA insertion in the first exon (Supplementary Fig. 5a), as confirmed by RT–PCR (Supplementary Fig. 5b). The single mutants were developmentally similar to Col-0 WT plants (Supplementary Fig. 5c) and did not differ from Col-0 WT in defence response to infection with *Pst* (EV) or *Pst* (*avrRpt2*) (Supplementary Fig. 5d). This lack of disease phenotype may be due to functional redundancy, so we generated the *arlpk1-1 arlpk2-1* double mutant. The *arlpk1-1 arlpk2-1* plants were smaller compared with Col-0 WT (Fig. 4a), and when infected with *Pst* (EV) and *Pst* (*avrRpt2*), *arlpk1-1 arlpk2-1* plants exhibited less bacterial growth in both cases (Fig. 4b). To confirm the phenotype of enhanced resistance in the double mutant, we measured the expression of antimicrobial pathogenesis-related protein, PR1, a marker for salicylic acid-dependent disease responses. At 12 h post inoculation (h.p.i.), *arlpk1-1 arlpk2-1* showed higher PR1 protein expression compared with Col-0 WT after infection with *Pst* (EV) and *Pst* (*avrRpt2*) (Fig. 4c), indicating that ARLPK1 and ARLPK2 are functionally redundant and negatively regulate plant immune responses.

We furthermore generated transgenic *Arabidopsis* overexpressing ARLPK1 with a mutation in the miR863-target site (mARLPK1-OE, as in Fig. 2a) in the Col-0 WT background. We selected two overexpression lines, mARLPK1-OE1 and -OE2, exhibiting high *ARLPK1* transcript levels as detected by real-time RT–PCR for further functional analysis (Fig. 4d). Both lines are more susceptible to *Pst* (EV) and *Pst* (*avrRpt2*) compared with Col-0 WT, further supporting that ARLPKs are negative regulators of plant immunity (Fig. 4e). miR863-3p-mediated silencing of *ARLPK1* during bacterial infection is important to promote plant defence responses.

### ARLPKs are putative pseudokinases

As noted above, ARLPK1 and ARLPK2 have variations in the conserved motifs of functional kinase domains such as the glycine-rich loop (GXGXXG), VAIK motif and HRD motif (Supplementary Fig. 3), and are thus predicted pseudokinases. The glycine loop is located in subdomain I near the catalytic domain and is found in many nucleotide-binding proteins as well as in protein kinases^25^. Both ARLPK1 and ARLPK2 have a serine substitution for the second glycine in the loop. The VAIK, HRD and DFG motifs are all found in the catalytic domain. The lysine in the VAIK motif in subdomain II interacts with ATP^25^. ARLPK1 and ARLPK2 have a VRVL and an IRVL motif, respectively, and are thus missing the lysine residue; it has not been demonstrated that these motifs can bind ATP. The aspartic acid in the HRD motif in subdomain IV is the base acceptor for proton transfer^25^. However, both pseudokinases have a HGN motif containing an uncharged asparagine. Finally, the aspartic acid in the DFG motif, which is found in catalytic domains and binds Mg^2+^ ions that coordinate the phosphates of ATP in the ATP-binding cleft^25^, is conserved in ARLPK1 and ARLPK2 (Supplementary Fig. 3). As predicted, we were unable to detect autophosphorylation *in vitro* using recombinant ARLPK1 and ARLPK2 proteins (Fig. 4f). In contrast, we were able to clearly detect kinase activity for the RPM1-induced protein kinase (RIPK), which was used as a positive control^30^. These data support that ARLPK1 and ARLPK2 are most likely pseudokinases.

### ARLPK1 interacts with AKIK1 to regulate plant immunity

**Figure 5:**
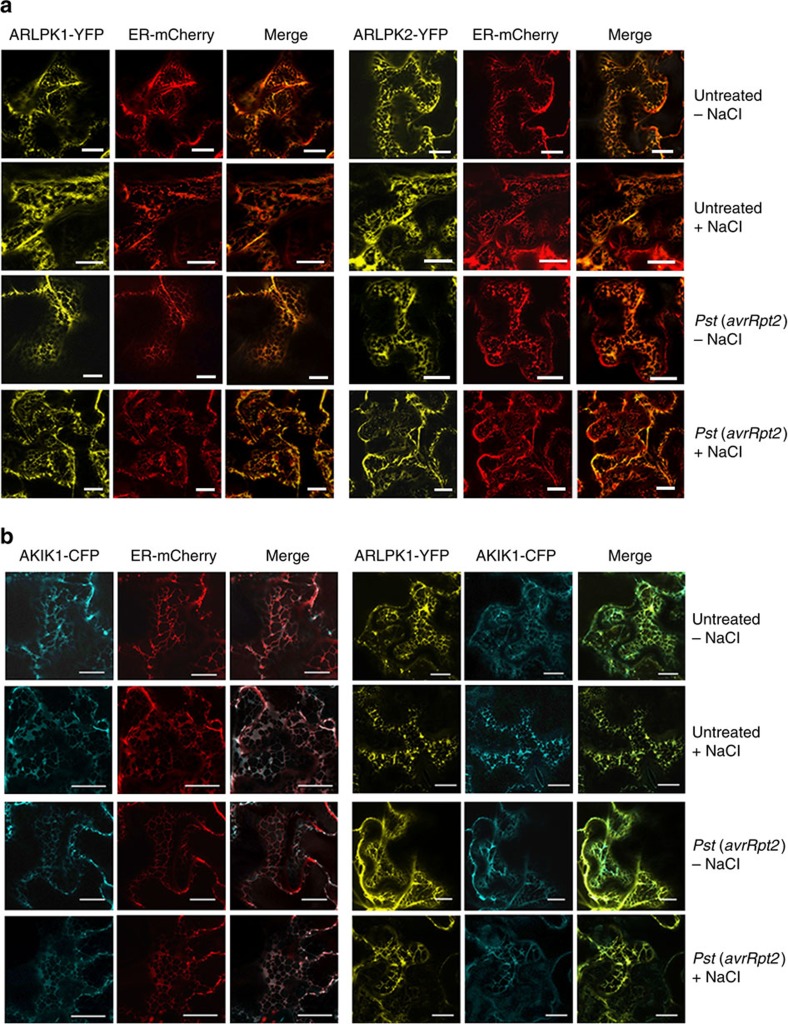
ARLPKs and AKIK1 predominantly localize in the ER. (**a**) Co-localization of ARLPK1-YFP or ARLPK2-YFP with ER-mCherry in *N. benthamiana* was observed with confocal microscopy. Untreated plants were visualized 60 h after co-infiltration. Treated plants were infected 48 h after co-infiltration with *Pst* (*avrRpt2*) and visualized 12 h.p.i. Bacterial inoculum concentration: 1 × 10^7^ c.f.u. per ml. Fluorescent images of the middle of the cell were taken separately then merged. Samples were visualized without (−NaCl) and with (+NaCl) plasmolysis treatment (5% NaCl for 5–10 min). Scale bars, 20 μm. (**b**) Co-localization of AKIK1-CFP with ER-mCherry and ARLPK1-YFP with AKIK1-CFP in *N. benthamiana* was observed with confocal microscopy. Untreated plants were visualized 60 h after co-infiltration. Treated plants were infected 48 h after co-infiltration with *Pst* (*avrRpt2*) and visualized 12 h.p.i. Tissue was collected for imaging 12 h.p.i. Bacterial inoculum concentration: 1 × 10^7^ c.f.u. per ml. Fluorescent images of the middle of the cell were taken separately then merged. Samples were visualized without (– NaCl) and with (+NaCl) plasmolysis treatment (5% NaCl for 5–10 min). Scale bars, 20 μm.

Both ARLPKs have a predicted transmembrane domain, so we determined their subcellular localization in *N. benthamiana* with confocal microscopy. However, the endoplasmic reticulum (ER) signal peptide in ARLPK1 and ARLPK2 overlaps with the predicted transmembrane domain (Supplementary Fig. 2). We originally thought that the pseudokinases would be localized in the plasma membrane (PM) and we used a PM-localized protein RLK (At5g23740)-cyan fluorescent protein (CFP) as control. To our surprise, we found that both ARLPK1-yellow fluorescent protein (YFP) and ARLPK2-YFP predominantly localize in the ER (Supplementary Fig. 6). We confirmed this using a mCherry-tagged ER organelle marker^31^ (ER-mCherry) as a control. ARLPK1-YFP and ARLPK-YFP overlapped with ER-mCherry in merged images, and Hechtian strands were not visible after plasmolysis treatment (Fig. 5a, upper panels). These results only show the steady-state localization of these fluorescently tagged proteins. Trafficking or movement of some RLKs during pathogen challenge is often observed, as in the case of FLS2, which undergoes endocytosis upon flg22 treatment^32^. We also checked the localization of ARLPKs after *Pst* (*avrRpt2*) challenge; however, we did not see any changes in the localization after *Pst* (*avrRpt2*) infection, both with and without plasmolysis treatment (Fig. 5a, lower panels).

Because these two kinases are putative pseudokinases that lack an extracellular domain, we hypothesized that ARLPK1 and ARLPK2 function with another RLK to perceive and/or regulate extracellular signals. We searched for interacting proteins on the Arabidopsis Interactions Viewer^33^ (http://bar.utoronto.ca/), and found AT5G59650, a LRR I subfamily RLK, which may interact with ARLPK1. Thus, we named it AKIK1. Owing to non-complementarity in the nucleotide alignment, AKIK1 is not likely to be targeted by miR863-3p. AKIK1 contains a malectin domain and two LRR domains in its predicted extracellular region (Supplementary Fig. 7a,b). We found that AKIK1-CFP also predominantly localizes in the ER in untreated and pathogen-treated plants, both with and without plasmolysis treatment, as seen in the merged image with ER-mCherry (Fig. 5b, left panels), and it also co-localizes with ARLPK1-YFP (Fig. 5b, right panels).

**Figure 6:**
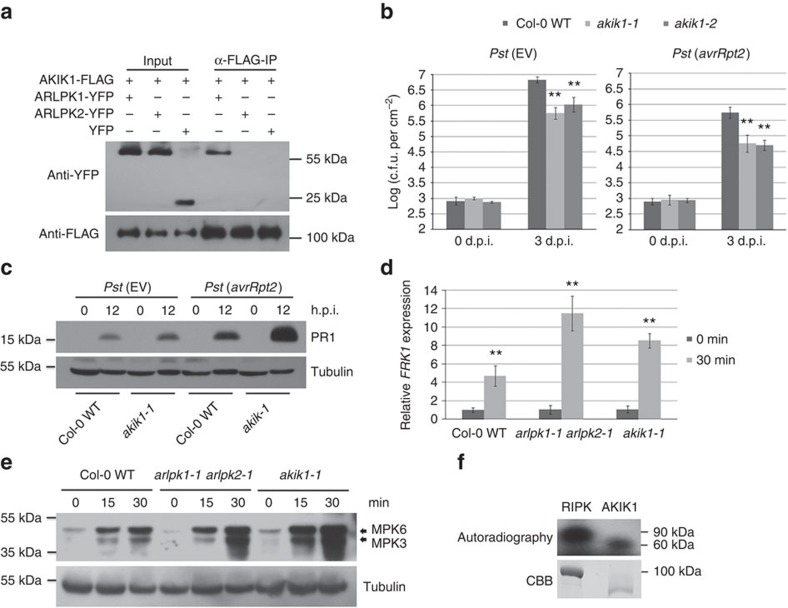
ARLPK1 interacts with AKIK1 that is also a negative regulator of disease resistance. (**a**) AKIK1-FLAG co-IP with ARLPK1-YFP but not with ARLPK2-YFP. Total proteins (input) from *N. benthamiana* extracts were immunoprecipitated with ANTI-FLAG M2 affinity gel, and AKIK1-FLAG and ARLPK1-YFP proteins were detected by western blot using anti-FLAG and anti-YFP antibodies, respectively. Similar results were obtained from three biological replicates. (**b**) The *akik1-1* and *akik1-2* mutants are more resistance to bacterial *Pst* (EV) and *Pst* (*avrRpt2*), as compared with WT plants. Bacterial inoculum concentration: 5 × 10^5^ c.f.u. per ml. Bacterial growth was measured 3 d.p.i. Error bars represent s.d. for at least 15 leaf discs. Similar results were obtained from three biological replicates. **P value <0.01 (Student's *t*-test). (**c**) PR1 protein levels in *Pst* (EV)- and *Pst* (*avrRpt2*)-infected Col-0 WT and *akik1-1* mutant were detected by western blot using an anti-PR1 antibody. Bacterial inoculum concentration: 1 × 10^7^ c.f.u. per ml. Leaf tissue was collected 0 and 12 h.p.i. α-Tubulin was used as a loading control. Similar results were obtained from two biological replicates. (**d**) Relative expression levels of *FRK1* in flg22-treated Col-0 WT, *arlpk1-1 arlpk2-1* and *akik1-1* mutants were detected by real-time RT–PCR. Samples were collected 0 and 30 min after treatment. Error bars indicate s.d. from three technical replicates. Similar results were obtained from three biological replicates. **P value <0.01 (Student's *t*-test). (**e**) The levels of phosphorylated MPK3 and MPK6 in flg22-treated Col-0 WT, *arlpk1-1 arlpk2-1* and *akik1-1* mutants were detected by western blot using monoclonal rabbit phospho-p44/42 MAPK (Erk1/2) (Thr202/Tyr204)(D13.14.4E) XP antibody. Leaf tissue was collected 0, 15 and 30 min after treatment. α-Tubulin was used as a loading control. Similar results were obtained from three biological replicates. (**f**) Recombinant MBP-tagged RIPK kinase domain and GST-tagged AKIK1 kinase domain were subjected to a radioactive kinase assay and kinase activity was detected using autoradiography. Bottom panel: SDS–PAGE gel stained with coomassie brilliant blue (CBB) demonstrating protein abundance. Similar results were obtained from two biological replicates.

We confirmed by co-immunoprecipitation (Co-IP) that ARLPK1, but not ARLPK2, interacts with AKIK1 (Fig. 6a). We hypothesized that due to its association with ARLPK1, AKIK1 also has a role in plant defence. We obtained two T-DNA insertion mutant lines, *akik1-1* (SALK_022711C) and *akik1-2* (CS848612), which have T-DNA insertions in the fourth and ninth introns of AKIK1, respectively (Supplementary Fig. 8a). These two lines are both null mutants of *AKIK1*, as confirmed by RT–PCR (Supplementary Fig. 8b), and neither had any obvious developmental phenotypes compared with Col-0 WT (Supplementary Fig. 8c). Both *akik1-1* and *akik1-2* were more resistant to infection with *Pst* (EV) and *Pst* (*avrRpt2*) compared with Col-0 WT plants (Fig. 6b). Because both mutants showed the same disease phenotypes, we used *akik1-1* for further analysis. In agreement with the bacterial growth assays, PR1 protein levels were higher in *akik1-1* compared with Col-0 WT 12 h.p.i. with *Pst* (EV) and *Pst* (*avrRpt2*), although to a lesser extent with *Pst* (EV) (Fig. 6c). To confirm the roles of ARLPK1, ARLPK2 and AKIK1 in PTI, we performed assays that measured PAMP responses to flg22 treatment. We measured PTI-responsive gene flg22-induced receptor-like kinase 1 (*FRK1*) by real-time RT–PCR after treatment with flg22. In agreement with the bacterial growth assay results, the double mutant *arlpk1-1 arlpk2-1* and the *akik1-1* mutant showed highly increased *FRK1* expression compared with Col-0 WT (Fig. 6d). Mitogen-activated protein kinase 3 (MPK3) and MPK6 are two kinases involved in the MAP kinase signalling cascade, which act downstream of FLS2 to confer disease resistance^34^. The *arlpk1-1 arlpk2-1* double mutant and the *akik1-1* mutant showed significantly higher MPK3 and MPK6 levels compared with Col-0 WT after flg22 treatment (Fig. 6e). Unlike ARLPK1 and ARLPK2, AKIK1 does possess conserved catalytic residues in its kinase domain (Supplementary Fig. 3). The kinase domain of AKIK1 was expressed and purified from *Escherichia coli* and subjected to an *in vitro* radiolabelled kinase assay. Consistent with its conserved catalytic residues, we were able to detect autophosphorylation activity for AKIK1, demonstrating that it is an active kinase (Fig. 6f). Taken together, the results show ARLPK1, ARLPK2 and AKIK1 all negatively regulate ETI and PTI.

### SE is a positive regulator of plant immunity

SE was originally identified as a C_2_H_2_-type zinc-finger protein important in leaf and meristem development, inflorescence architecture and phase transition^28^,^35^. Since then, it has been shown to play a role in a crucial step of miRNA maturation and accumulation^18^,^19^,^20^,^21^, pre-mRNA splicing^36^ and alternative splicing^24^. We hypothesized that as a target of miR863, *SE* should have a role in disease resistance; however, nothing has been uncovered so far regarding its function in antibacterial defence in plants.

**Figure 7:**
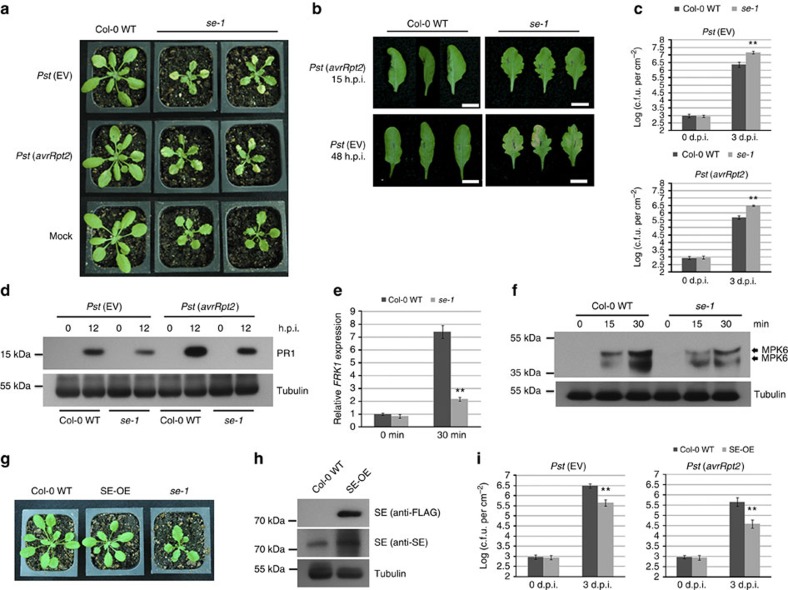
SE is a positive regulator of disease resistance. (**a**) The *se-1* mutant displays enhanced disease susceptibility phenotype after *Pst* (EV) and *Pst* (*avrRpt2*) infection. Bacterial inoculum concentration: 5 × 10^5^ c.f.u. per ml. Photos were taken 3 d.p.i. Mock-treated plants were used as controls. (**b**) The *se-1* mutant shows delayed HR after *Pst* (*avrRpt2*) infection, and enhanced disease phenotype after *Pst* (EV) infection. Pictures were taken 15 h.p.i. of *Pst* (*avrRpt2*) infection and 48 h.p.i. of *Pst* (EV) infection. In all, 16 out of 18 *Pst* (*avrRpt2*)-infected Col-0 WT leaves exhibited HR at 15 h.p.i. under our conditions (three with HR are shown), while only 3 out of 18 of the *se-1* leaves showed HR at 16 h.p.i. (three without HR are shown). A total of 12 out of 15 *Pst* (*avrRpt2*)-infected *se-1* leaves exhibited chlorosis, while only 3 out of 15 Col-0 WT leaves did. Only half of the leaf was inoculated. Bacterial inoculum concentration: 1 × 10^7^ c.f.u. per ml. Scale bar, 10 mm. (**c**) The *se-1* mutant is more susceptible to the infection of *Pst* (EV) and *Pst* (*avrRpt2*) than Col-0 WT. Bacterial inoculum concentration: 5 × 10^5^ c.f.u. per ml. Bacterial growth was measured 3 d.p.i. Error bars represent s.d. for at least 15 leaf discs. Similar results were obtained from three biological replicates. **P value <0.01 (Student's *t*-test). (**d**) PR1 protein levels in *Pst* (EV)- and *Pst* (*avrRpt2*)-infected Col-0 WT and *se-1* mutants were detected by western blot using an anti-PR1 antibody. Bacterial inoculum concentration: 1 × 10^7^ c.f.u. per ml. Leaf tissue was collected 0 and 12 h.p.i. α-Tubulin was used as a loading control. Similar results were obtained from two biological replicates. (**e**) Relative expression levels of *FRK1* in flg22-treated Col-0 WT and *se-1* mutant were detected by real-time RT–PCR. Leaf tissue was collected 0 and 30 min after treatment. Error bars indicate s.d. from three technical replicates. Similar results were obtained in three biological replicates. **P value <0.01 (Student's *t*-test). (**f**) MPK3 and MPK6 protein levels in flg22-treated Col-0 WT and *se-1* mutant were detected by western blot using monoclonal rabbit phospho-p44/42 MAPK (Erk1/2) (Thr202/Tyr204)(D13.14.4E) XP antibody. Samples were collected 0, 15 and 30 min after treatment. α-Tubulin was used as a loading control. Similar results were obtained from two biological replicates. (**g**) Phenotypes of 4-week-old FLAG-tagged SE overexpression lines (SE-OE), *se-1* mutants and Col-0 WT plants. (**h**) SE protein levels were detected in Col-0 WT and FLAG-tagged SE-OE lines by western blot using anti-FLAG and anti-SE antibodies. α-Tubulin was used as a loading control. Similar results were obtained from two biological replicates. (**i**) Bacterial growth in *Pst* (EV)- and *Pst* (*avrRpt2*)-infected Col-0 WT and SE-OE lines. Bacterial inoculum concentration: 5 × 10^5^ c.f.u. per ml. Bacterial growth was measured 3 d.p.i. Error bars represent s.d. for at least 15 leaf discs. Similar results were obtained from three biological replicates. **P value <0.01 (Student's *t*-test).

We obtained the *se-1* mutant (CS3257), which contains an X-ray-induced 7-base deletion, for analysis^21^,^28^,^35^. The *se-1* mutant is smaller, has serrated leaves and exhibits pleotropic defects in shoot and leaf development^28^. We inoculated the *se-1* plants with *Pst* (EV), *Pst* (*avrRpt2*) and a mock solution to determine the role of SE in disease resistance. The *se-1* mutant showed increased disease susceptibility manifested as stronger chlorosis, or yellowing of the leaves, 3–4 d.p.i. (days post inoculation) compared with Col-0 WT plants. Infection with pathogen does not stunt growth of *se-1* mutants (Fig. 7a). The *se-1* mutant also showed delayed HR after infection with *Pst* (*avrRpt2*), indicating a weaker or slower RPS2-mediated defence response against *Pst* (*avrRpt2*) (Fig. 7b). We included *Pst* (EV) infection as a control, which did not trigger HR; however, at 48 h.p.i., the *se-1* mutant showed chlorosis of the leaves (Fig. 7b) while Col-0 WT did not. We also measured the bacterial titre in *se-1* mutant and found that both *Pst* (EV) and *Pst* (*avrRpt2*) growth was greater in the mutants compared with Col-0 WT plants (Fig. 7c). Consistent with the bacterial growth assays, PR1 protein expression is lower in *se-1* mutant than in Col-0 WT after infection with *Pst* (EV) and *Pst* (*avrRpt2*) (Fig. 7d). *FRK1* transcript level (Fig. 7e) and MPK6 and MPK3 protein levels (Fig. 7f) are lower in *se-1* after flg22 treatment than in Col-0 WT. Furthermore, we obtained *Arabidopsis* transgenic lines overexpressing *SE* CDS in the *se-1* background^21^,^24^ (SE-OE) that have a complemented leaf phenotype and are slightly smaller than Col-0 WT plants (Fig. 7g). We confirmed the overexpression of *SE* in these lines (Fig. 7h), and found that they show enhanced disease resistance to *Pst* (EV) and *Pst* (*avrRpt2*) (Fig. 7i). Taken together, these data further support SE is a positive regulator of disease resistance.

### miR863 sequentially silences its targets to regulate defence

**Figure 8:**
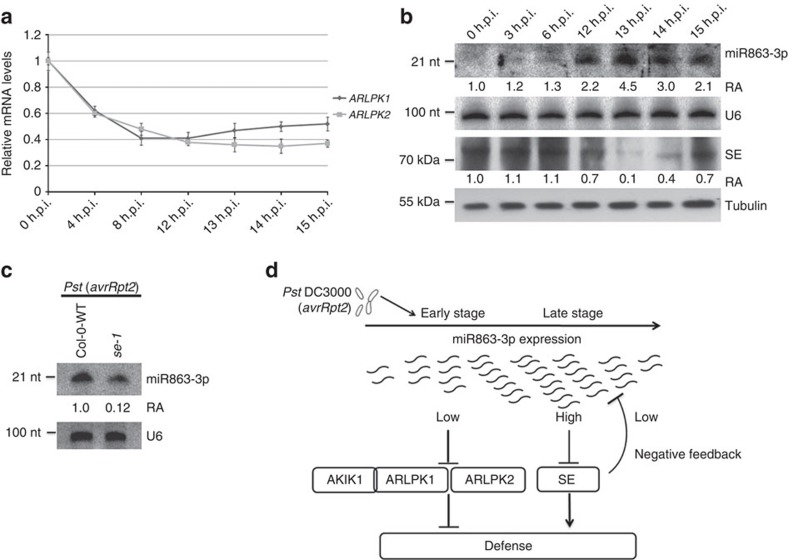
miR863-3p regulates ARLPK1, ARLPK2 and SE in a time-dependent manner. (**a**) Time course of relative expression levels of *ARLPK1* and *ARLPK2* transcripts in *Pst* (*avrRpt2*)-infected Col-0 WT plants were detected by real-time RT–PCR. Bacterial inoculum concentration: 5 × 10^6^ c.f.u. per ml. Error bars indicate s.d. from three technical replicates. Similar results were obtained in three biological replicates. **P value <0.01 (Student's *t*-test). (**b**) Time course of miR863-3p levels in *Pst* (*avrRpt2*)-infected Col-0 WT plants was detected by northern blot analysis. U6 was used as a loading control. SE protein levels in *Pst* (*avrRpt2*)-inoculated Col-0 WT plants were detected by western blot analysis using an anti-SE antibody. α-Tubulin was used as a loading control. Relative abundance (RA) levels are indicated. Bacterial inoculum concentration: 5 × 10^6^ c.f.u. per ml. Similar results were obtained from two biological replicates. (**c**) miR863-3p levels in *Pst* (*avrRpt2*)-infected Col-0 WT and *se-1* mutants were detected by northern blot analysis. U6 was used as a loading control. Bacterial inoculum concentration: 1 × 10^7^ c.f.u. per ml. Samples were collected 14 h.p.i. RA levels are indicated. Similar results were obtained from three biological replicates. (**d**) A model of miRNA863-3p downregulation of targets after infection with *Pst* (*avrRpt2*). During the early stage of infection, miR863-3p levels are upregulated and suppress ARLPK1 and ARLPK2 to enhance disease resistance. ARLPK1 interacts with LRR-RLK AKIK1 to negatively regulate defence. During the later stage of infection, miR863-3p downregulates SE. Downregulation of SE results in a decrease in miR863-3p levels, forming a negative feedback loop to suppress defence responses.

Our results showed that the three confirmed targets of miR863 have opposite roles in plant immunity. While the ARLPKs are negative regulators of defence, SE is a positive regulator, thus explaining why overexpressing miR863 has no clear effect on disease resistance (Fig. 3e). Thus far, known miRNAs involved in immunity target either negative or positive regulators of plant immunity, not both. To find out why miR863-3p silences targets with opposite cellular functions, we performed a time course experiment and examined the levels of the targets during early and late stages after infection with *Pst* (*avrRpt2*). Because high inoculum concentration of avirulent *Pst* can trigger HR and thus affect the expression levels of many genes, we used a lower concentration (2 × 10^6^ colony-forming units (c.f.u.) per ml) that would not trigger macroscopic HR at 15 h.p.i., but could still induce miR863-3p levels. Real-time RT–PCR and western blot analysis revealed the kinases are downregulated early, just at 4 h.p.i. (Fig. 8a), while SE protein levels are downregulated much later, around 12 h.p.i. (Fig. 8b). It may be that SE is not targeted by miR863 during earlier stages of infection because a higher level of miR863 is required for translation inhibition of SE, which is not achieved until the late stage of infection. After SE protein levels decrease, the accumulation of miR863-3p also decreases in the later stages of infection (Fig. 8c). We probed miR393 and miR319 as well and found that the levels of both also decrease in latter stages of infection similar to that of miR863-3p, showing that downregulation of SE protein does indeed affect miRNA accumulation (Supplementary Fig. 9a). We also performed the time course experiment with *Pst* (EV) infection and found that the levels of *ARLPK1* and *ARLPK2* mRNA, miR863-3p and SE protein did not significantly change over time (Supplementary Fig. 9b). These results show that miR863-3p temporal regulation of its targets is more predominant in ETI, although ARLPK1, ARLPK2 and AKIK1 all have roles in negative regulation of both ETI and PTI.

## Discussion

The initiation, timing and amplitude of plant defence responses are tightly controlled. Cellular reprogramming that occurs during pathogen infection is energy consuming; thus, a trade-off exists between plant growth and defence^37^. In recent years, several miRNAs and siRNAs with important roles in plant immunity have been discovered. However, no sRNAs that target genes with antagonistic roles in response to the same environmental or developmental cues have been reported in any cellular processes within any organisms. Here we identified miR863-3p, which is highly induced by *Pst* (*avrRpt2*) infection and sequentially silences negative and positive regulators of plant immunity through two different modes of action. miR863-3p first suppresses negative regulators—ARLPK1 and ARLPK2—via mRNA degradation to boost defence responses quickly after infection. ARLPK1 interacts with AKIK1, which also functions as a negative regulator. Then, during later stages of infection, miR863-3p downregulates SE via translational inhibition to attenuate defence signalling by bringing down the level of miR863-3p, of which accumulation is dependent on SE (Fig. 8c,d), thus forming a negative feedback loop to attenuate plant immunity. Thus, miR863-3p plays a critical role in regulating and fine-tuning the timely upregulation of plant defences on pathogen infection, as well as the attenuation of immune responses after successful defence. Just as the timely upregulation of plant defences on pathogen infection is crucial for survival, the attenuation of immune responses after successful defence may also be important to conserve host resources for growth and reproduction.

Even though miR863-3p regulates ARLPK1, ARLPK2 and SE during ETI, all targets, as well as AKIK1, are regulators of not only ETI but also PTI. A recent systematic study of network characteristics in ETI and PTI revealed that both share a large subnetwork of genes and interactors that most likely functions to favour plant defence over growth^38^. However, the organization of defence response modules in ETI is independent, rather than cohesive as it is in PTI, due to the evolutionary demand for a rapid and robust response to pathogen effectors^38^.

The RLK/Pelle family has over 600 members and is the largest group of protein kinases in *Arabidopsis*^23^. Many RLKs are extensively involved in signalling pathways in development and plant immunity^39^,^40^,^41^. In the recent years, several RLKs that play a role in negative regulation of plant defence have been discovered, such as growth-promoting phytosulfokine (PSK) receptors PSKR1 and PSY1R (refs 42, 43), and BAK1-interactors BIR1 (ref. 44) and BIR2 (ref. 45). Interestingly, the BIR2 kinase domain is missing conserved motifs and exhibits no kinase activity^45^. Nearly 20% of *Arabidopsis* RLKs are predicted to be pseudokinases and can have phosphorylation-independent mechanisms of mediating signal transduction^46^. Many pseudokinases have important functions in humans^47^, plants^48^,^49^,^50^,^51^ and bacteria^52^,^53^, and still possess kinase catalytic activity or bind nucleotides and/or cations. They can act as scaffolding proteins or regulate the activity of functional kinases^25^. Neither ARLPK1 nor ARLPK2 exhibited detectable autophosphorylation *in vitro* (Fig. 4f) and may thus function as scaffolding proteins or modulate the activity of AKIK1 or other kinases to regulate immune signalling.

Most studied RLKs, including PSKY1 (ref. 54), PSY1R (ref. 55), BIR1 (ref. 44) and BIR2 (ref. 45), localize in the plasma membrane, but we found that ARLPK1, ARLPK2 and AKIK1 were all predominantly localized to the ER (Fig. 5 and Supplementary Fig. 6). ARLPK1 and ARLPK2 both have predicted signal peptides that overlap with their transmembrane domains (Supplementary Fig. 2); thus, it is possible that the sequences are not true signal peptides. In contrast, AKIK1 has a clear signal peptide (Supplementary Fig. 7). It is possible that AKIK1 also localizes in other cellular organelles. Future experiments would involve obtaining native antibodies specific to AKIK1, ARLPK or ARLPK2, to determine their subcellular localization *in vivo*. The multigene family of hormone ethylene receptor kinases—including ETR1, ERS1, CTR1 and EIN2—are ER-membrane bound. Ethylene plays a role in many plant growth and development processes, as well as responses to environmental stresses, and its gaseous nature may explain why its receptors can localize in the ER^56^. *Arabidopsis* hormone cytokinin receptors—AHK2, AHK3 and CRE1/AHK4—which play roles in plant growth and formation, as well as in biotic and abiotic stress responses, are all membrane-bound kinases that mainly localize in the ER. These receptors may be localized in the ER for crosstalk or for close proximity to the nucleus for downstream signalling^57^. On the other hand, *Arabidopsis* AtIre1-1 and AtIre1-2 are transmembrane receptor kinases located in the ER and most likely function in ER unfolded protein response signalling, like their yeast and mammalian homologue Ire1 (ref. 58). How the roles of ARLPK1, ARLPK2 and AKIK1 are related to their subcellular localization in the ER remains to be determined. Finding their downstream components or other interacting partners may shed light on this question.

The role of SE in antibacterial plant defence has not been previously uncovered. However, *dcl1*, *hyl1* and *hen1*, the mutants defective in miRNA accumulation, have decreased basal defence^12^,^14^. One study showed that SE might be involved in the cuticle integrity pathway, and related to that, *se-1* mutants are more susceptible to necrotrophic fungal infection^59^. SE has been proposed to regulate gene expression by modifying chromatin structure^35^. As a component of the nuclear cap-binding complex, SE also has a role in pre-mRNA splicing^36^. This distinguishes SE from DCL1 and HYL1, which participate in only miRNA processing^24^. Other SE-dependent miRNAs are also likely to be involved during later infection stages to returning the host immune system to a normal state. Future research focusing on the role of additional SE-related miRNAs as well as ARLPK1 and ARLPK2 targets will enhance our understanding of temporal immune signalling.

## Methods

### Plant materials and growth conditions

*Arabidopsis* and *N. benthamiana* plants were grown in a controlled growth room at 23±1 °C in a 12-h light/12-h dark photoperiod. All experiments were performed on 4-week-old *Arabidopsis* plants and 3-week-old *N. benthamiana* plants. The following mutants seeds were used in this study: *dcl1-7/fwf2* (ref. 13), *rdr6-15* (ref. 60), *se-1* (ref. 28), and SALK lines *arlpk-1* (SALK_144635), *arlpk-1* (SALK_040744), *akik1-1* (SALK_022711C) and *akik1-2* (CS848612)^29^. Homozygous mutants plants were identified by RT–PCR, and primers are listed in Supplementary Table 1. All mutants were in the Columbia (Col-0) background, except for *dcl1-7/fwf2*, which was in the Landsberg *erecta* (L*er*) background.

### Identification of target proteins of miR863

To identify miR863-3p target genes, a set of computational rules for target prediction was adapted from Allen *et al*.^61^ and modified. The miRNA-target duplex must contain four or less unpaired bases, four or less G:U pairs, up to one single-nucleotide bulge and seven or fewer unpaired plus G:U positions. Nucleotides at positions 10 and 11 of the miRNA must be a perfect match with its target. Mismatched pairs or single-nucleotide bulges were given a score of 1; G:U pairs were given a score of 0.5. Mismatched and G:U pair scores were doubled if they were located within the core segment (positions 2–13). A maximum of three continuous mismatches was allowed if the mismatch region contained at least two G:U pairs, and the penalty score of the region was multiplied by 1.5. We used 5.5 as the cutoff score for selecting the miRNA targets.

### Bacterial infection

Bacterial strains used include the following: *Pseudomonas syringae* pv. *tomato* DC3000 carrying broad host range vector pVSP61 (ref. 62) (EV); or pVSP61 plasmid containing avirulence gene *avrRpt2* (ref. 62), *avrRpm1* (ref. 63) or *avrRps4* (ref. 64); and a strain that has a mutation in its type III secretion system (Δ*hrcC*)^65^. For bacterial growth assays, 4-week-old *Arabidopsis* plants were syringe-infiltrated with a 5 × 10^5^ c.f.u. per ml bacterial suspension. Leaves were collected 3 d.p.i. using a cork borer, and bacterial titre was determined by serial dilution, plating and counting the colonies. At least 15 leaf discs were collected for each growth assay. Three biological repeats were performed with similar results. Student's *t*-test was used to determine the significant differences between mutants and control plants. For northern and western blots and the HR assay, a 1 × 10^7^ c.f.u. per ml bacterial suspension was used. A total of 18 leaves were infected for the HR assay and monitored for the appearance of HR symptoms. For the time course, a 2 × 10^6^ c.f.u. per ml bacterial suspension was used.

### Generation of transgenic plants

To generate the construct for the MIR863-OE lines, the miR863 precursor was cloned using a miR319 backbone^66^ into a pEarleyGate (pEG) 100 destination vector^67^ using LR clonase II (Invitrogen). To generate the mARLPK1-OE lines, the mutated (m) ARLPK1 CDS was cloned into pEG104. *Arabidopsis* plants were transformed using floral dip method with *Agrobacterium tumefaciens* strain GV3101 carrying the carrying cloned vectors.

### Protein extraction and analysis

Tissue sample was ground in liquid nitrogen and total proteins were extracted using 2 × SDS loading buffer. The samples were resolved on a 12% SDS–PAGE gel and transferred onto nitrocellulose membranes using a Tris-Glycine transfer buffer. The blots were probed with the appropriate antibodies: monoclonal mouse anti-GFP, which also recognizes YFP (Roche, 11814460001, 1:2,000 dilution); monoclonal mouse anti-FLAG (Sigma-Aldrich, F3165, 1:2,000 dilution); monoclonal mouse anti-α tubulin (Sigma-Aldrich, T6074, 1:4,000 dilution); polyclonal rabbit anti-PR1 (obtained from Xinnian Dong^68^, 1:2,000 dilution); polyclonal rabbit anti-SE (serum containing polyclonal antibodies was produced in rabbits immunized with peptide containing the first 200 amino acids of the SE protein, AbMax Biotechnology Co., Ltd., 1:1,000 dilution); rabbit serum was purified using Montage Antibody Purification kit, Millipore); goat anti-mouse IgG-HRP (Santa Cruz Biotechnology, sc-2005, 1:4,000 dilution); and goat anti-rabbit IgG-HRP (Santa Cruz Biotechnology, sc-2030. 1:4,000 dilution).

### Transient expression analysis in *N. benthamiana*

Transient co-expression and co-immunoprecipitation assays in *N. benthamiana* were performed by infiltrating 3-week-old *N. benthamiana* plants with *Agrobacterium* (OD_600_ (optical density at 600 nm)=1.0) harbouring constructs containing the miR863 precursor with *Agrobacterium* containing (OD_600_=1.0) wtARLPK1 or mutated (m) ARLPK1 CDS (pEG104), wtARLPK2 or mARLPK2 CDS (pEG104), and GFP-wtSE-3′-UTR or GFP-mSE-3′-UTR (pEG103). Leaf tissue was collected 48 h.p.i. and processed as described above. AKIK1 (pEG202) was used for Co-IP assays with ARLPK1 and ARLPK2. ANTI-FLAG M2 affinity gel (Sigma-Aldrich, A2220) was used for pull downs.

### 5′-RACE of mRNA cleavage products

5′-RACE^69^ was performed using FirstChoice RLM-5′-RACE kit (Ambion) following the manufacturer's instructions. Briefly, total RNA was extracted from *Pst* (*avrRpt2*)-infected tissue 10 h.p.i. and directly ligated to the RNA Oligo adaptor without further modification. Oligo (dT) primer was used to prime cDNA synthesis with reverse transcriptase. Gene-specific 5′-RACE reactions were done with the 5′ Nested Primer and gene-specific primers ARLPK1-R 5′-RACE and ARLPK2-R 5′-RACE as listed in Supplementary Table 1. The 5′-RACE products were gel purified, cloned into pENTR (Invitrogen) vector and sequenced.

### Site-directed mutagenesis

The wtARLPK1 CDS, ARLPK2 CDS and SE 3′-UTR sequences were cloned into pENTR (Invitrogen) vector. Point mutations were introduced using the GeneArt Site-Directed Mutagenesis System kit (Invitrogen) following the manufacturer's instructions. Primers used are listed in Supplementary Table 1 as mARLPK1-F, mARLPK1-R, mARLPK2-F, mARLPK2-R, mSE 3′-UTR-F and mSE 3′-UTR-R. The final plasmids with the mutated sequences were cloned into the pEG104 (mARLPK1 and mARLPK2) or pEG103 (mSE-3′-UTR) destination vectors using LR Clonase II (Invitrogen).

### Protein kinase assays

ARLPK1 and ARLPK2 were cloned into pMAL-C4X with N-terminal fusions to maltose-binding protein (MBP). MBP-ARLPK1, MBP-ARLPK2 and MBP-RIPK were induced with 0.3 mM isopropyl-β-D-thiogalactoside for 3 h at 28 °C and purified by amylose affinity chromatography from *E. coli*. The kinase domain of AKIK1 (547–892 aa) was cloned into pDEST-15 (Invitrogen) with an N-terminal fusion to glutathione *S*-transferase (GST). GST-AKIK1 was expressed in *E. coli* (BL21 strain) at 28 °C for 4 h and the recombinant protein purified with Glutathione Sepharose 4B (GE Healthcare). Kinase assays were performed using 3 μg of recombinant protein with [γ-^32^P]-ATP. The assay was initiated by adding 1 ml (10 μCi) ^32^P-ATP and incubated for 40 min at 30 °C. The reaction was terminated by the addition of 3 × laemmli loading buffer and subsequent incubation at 95 °C for 5 min. The proteins were separated on a 12% SDS–PAGE gel and signals were visualized by X-ray film exposure^30^.

### RNA extraction and analysis

Fresh tissue was ground in liquid nitrogen and RNA was extracted using TRIzol Reagent (Invitrogen) following the manufacturer's instructions. RNA is resolved on a 17% denaturing 8 M urea-PAGE gel and then transferred and chemically crosslinked onto a Hybond N^+^ membrane (GE Healthcare Life Sciences) using *N*-(3-Dimethylaminopropyl)-*N*′(3-Dimethylaminopr hydrochloride. miRNAs were detected using end-labelled oligonucleotide probes and exposed to a phosphor imager screen. ImageQuant TL 7.0 analysis software (GE Healthcare Life Sciences) was used to measure relative abundance levels. For quantification of relative gene expression, cDNA was synthesized using Superscript III (Invitrogen), and real-time RT–PCR was performed using SYBR green dye (Bio-Rad) on a MyiQ detection system (Bio-Rad). The primers for all experiments are listed in Supplementary Table 1.

### MAPK activity assay

Two-week-old seedlings grown on half-strength Murashige and Skoog medium were treated with 10 μM flg22 containing 0.01% Silwet L-77. Samples were collected at 0, 5, 10, 15 and 30 min, and analysed by western blotting using monoclonal rabbit phospho-p44/42 MAPK (Erk1/2) (Thr202/Tyr204)(D13.14.4E) XP antibodies (Cell Signaling Technology, #4370S, 1:2,000 dilution). α-tubulin was used as a loading control using monoclonal mouse anti-α tubulin (Sigma-Aldrich, T6074, 1:4,000 dilution).

### Subcellular localization

Subcellular localization of fluorescent-tagged proteins was determined using confocal microscopy. Three-week-old *N. benthamiana* plants were infiltrated with *Agrobacterium* carrying constructs ARLPK1-YFP, ARLPK2-YFP, AKIK1-CFP, RLK-CFP, ER-mCherry (ER-rk; *CD3-959*) or empty GFP vector. Untreated tissue was collected 48 h.p.i. Plants were treated with *Pst* (*avrRpt2*) 48 h.p.i. after infiltration with *Agrobacterium* and tissue was collected 12 h.p.i. For the plasmolysis treatment, tissue was treated with 5% NaCl for 5–10 min before visualization (+NaCl), or with water as a control (–NaCl). The ER marker is the mCherry fluorescent protein with the signal peptide of *Arabidopsis* wall-associated kinase 2 (AtWAK2) at the N terminus and the ER retention signal His-Asp-Glu at the C terminus^31^. The plasma membrane marker is an RLK (At4g23740) fused to CFP at the C terminus^70^.

Full uncropped versions of each gel and blot image are included in Supplementary Fig. 10. Results for biological replicates are included in Supplementary Fig. 11.

## Additional information

**How to cite this article:** Niu, D. *et al*. miRNA863-3p sequentially targets negative immune regulator *ARLPKs* and positive regulator *SERRATE* on bacterial infection. *Nat. Commun.* 7:11324 doi: 10.1038/ncomms11324 (2016).

## Supplementary Material

Supplementary Information Supplementary Figures 1-11, Supplementary Table 1 and Supplementary References
