# Supplementary material for: miRNA863-3p sequentially targets negative immune regulator ARLPKs and positive regulator SERRATE upon bacterial infection
Source: Nat Commun. 2016 Apr 25;7:11324. doi: 10.1038/ncomms11324 (PMC4848489; doi:10.1038/ncomms11324)
Supplement: Supplementary Information — Supplementary Figures 1-11, Supplementary Table 1 and Supplementary References [file ncomms11324-s1.pdf]

Supplementary Figures and Legends (JIN)

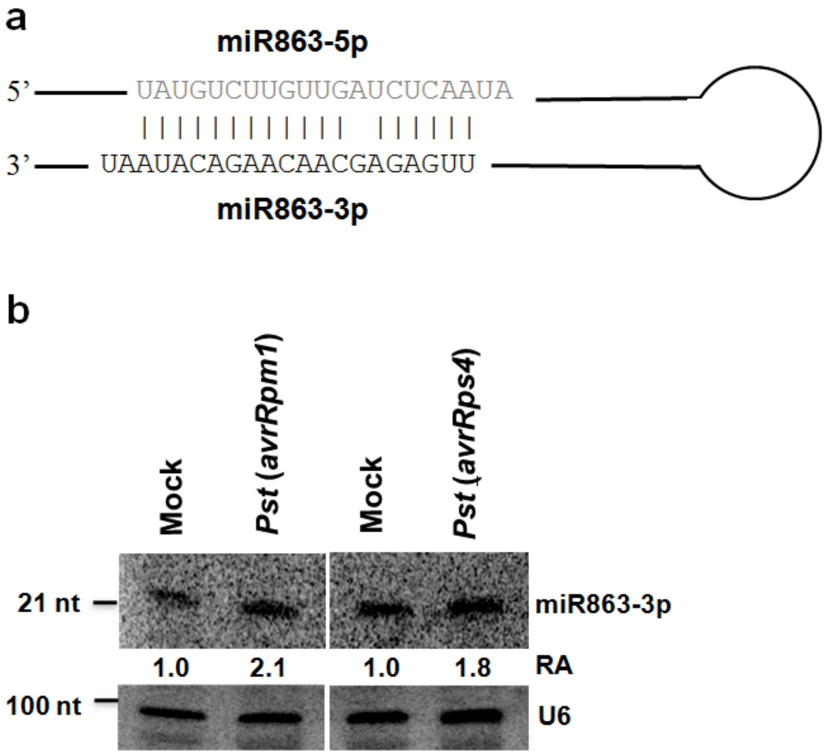

Supplementary Figure 1. miR863-3p is induced by bacterial infection.

(a) miR863 precursor fold-back structure containing miR863-3p and -5p with 2' overhang.

(b) miR863-3p levels in mock-, *Pst* (*avrRpm1*)- and *Pst* (*avrRps4*)-infected Col-0 WT plants were detected by Northern blot. Bacterial inoculum concentration: 1 x 10^7 CFU/ml. *Pst* (*avrRpm1*)- *Pst* (*avrRps4*)-infected leaf tissue was collected at 8 hpi and 24 hpi, respectively. U6 was used as a loading control. Relative abundance (RA) levels are indicated.

a

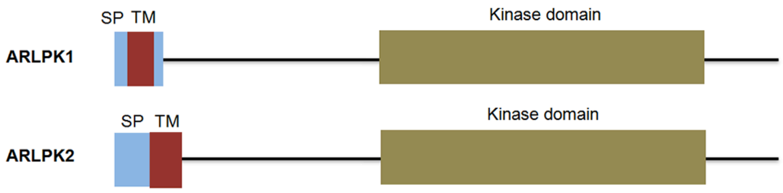

b

ARLPK1

Signal peptide Transmembrane domain

MRKSHLVKLILAVSLPTTFLIMIAIIIIFIFCRRTTTETNEVQYDVESPYEKQEFSDNGSETEEEL
IIFNGGEDLTICDILDAPGEVIGKSSYGTLYKATLQRSGKVRVLRFLRPLCAVNSDSKEFNGVI
ESLGFVRHDNLVPLLGFYVGNRGEKLMIHPFFGSSGNLSAFIKFLAGGDVDAHKWSNILSITI
GIAKALDHLHTGMQKPIVHGNLKSKNVLLDKSFRPRVSDFGLHLLLNLAAGQEVLEASAA
EGYKAPELIKMKEVSKESDVYSFGVIMLELVSGKEPTNKNPTGSVLDRNRLSDLYRPEIIRR
CLKDGNGVTEECVLEYFQLAMSCCSPSPTLRPSFKQVLRKLEEIRK

Kinase domain

ARLPK2

Signal peptide Transmembrane domain

MRKSLLLTLLLGVSLSAFLIVIFFIIFLRRKESSSTESDQYDVESLDHNKQGFSSETEELVIFQG
GEDLTICDILDAPGEVIGKSSYGTLYKASLQRSGKIRVLRFLRPVCTVRSDSKEFNGIIETLVR
HENLVPLLGFYAGNRGEKLMVHPFFGSGNLSDFIRSGDDESRKWINILRITIGISKALDHLHT
GMQKPIVHGNLKSKNVLLSSSFEPRISDFGLHLLLNLSAGQEILDVSAAEGYKAPELIKMKD
VSKESDVYSLGVIMLELVSGKEPINENATGDDEFYLPDFMRNAVLDHRLSDLYRPEILGSDD
NLSEECVLKYFQLAMSCCSPSPSLRPNVKQVLRKLEEIGKF

Kinase domain

Supplementary Figure 2. ARLPK1 and ARLPK2 are atypical receptor-like kinases lacking extracellular domains.

(a) Diagrams of ARLPK1 and ARLPK2 with important predicted domains indicated. SP (blue box): signal peptide; TM (red box): transmembrane domain; kinase domain (brown box)

(b) Amino acid sequences of ARLPK1 and ARLPK2 with signal peptide (blue), transmembrane domain (highlighted in yellow), and kinase domain (brown) indicated.

```
                         I                            II                           III
ARLPK1 ILDAPGEVIGKSSYGTLYKATLQRSGKVRVLRFLRPLCAVNSDSK-EFNGVIESLG  134
ARLPK2 ILDAPGEVIGKSSYGTLYKASLQRSGKIRVLRFLRPVCTVRSDSK-EFNGIIETLG  128
AKIK1  MTNNFQRVVGEGGFGVVCHGTINGSEQVAVKVLSQSSSQGYKHFKAEVDLLL----  636
                    GxGxxG                 VAIKxL                E
                   IV                               V
ARLPK1 FVRHDNLVPLLGFYVGNRGEKLMIHPFFGSSGNLSAFIKCGDVDAHKWSNILSITI  190
ARLPK2 FVRHENLVPLLGFYAGNRGEKLMVHPFFGS-GNLSDFIRSGDDESRKWINILRITI  183
AKIK1  RVHHTNLVSLVGYCDERDHLALIYEFLPKGDLRQHLSGKSGGSFIN-WGNRLRIAL  691
              L

                           VI                            VII
ARLPK1 ----GIAKALDHLHTGMQKPIVHGNLKSKNVLLDKSFRPRVSDFGLHLLLNLAAGQ  242
ARLPK2 ----GISKALDHLHTGMQKPIVHGNLKSKNVLLSSSFEPRISDFGLHLLLNLSAGQ  235
AKIK1  EAALGLEYLHSGCTPPIV----HRDIKTTNILLDEQLKAKLADFGLSRSFPI--GG  741
          G--YL              HRDLKxxN           DFG
                          VIII          IX
ARLPK1 EVLEAS---AAE-GYKAPELIKMKEVSKESDVYSFGVIMLELVSGKEPTNKNPTG-  293
ARLPK2 EILDVS---AAE-GYKAPELIKMKDVSKESDVYSLGVIMLELVSGKEPINENATGD  287
AKIK1  ETHI-STVVAGTPGYLDPEYYQTTRLGEKSDVYSFGIVLLEIITNQPVIDQSRSK-  795
                         APE           D-YS-G
                                     X
ARLPK1 KEPTNKNPTG-----------SVLDRNRLSDLYRPEIIRRCLKDGNG-VTEECVLE  328
ARLPK2 KEPINENATGDDEFYLPDFMRNAVLDHRLSDLYRPEILG---SDDN--LSEECVLK  327
AKIK1  ---------------------SHISQWVGFELTRGDITKIMDPNLNGDYESRSVWR  830

                 XI
ARLPK1 YFQLAMSCCSPSPTLRPSFKQVLRKLEEIRK                           358
ARLPK2 YFQLAMSCCSPSPSLRPNVKQVLRKLEEIGKF                          359
AKIK1  VLELAMSCANPSSVNRPNMSQVANELKECLVS                          880
             M---------R
```

Supplementary Figure 3. Amino acid alignments of ARLPK1, ARLPK2, and AKIK1 kinase domains.

Alignments were made using Clustal Omega (1.2.1)[1]. Consensus sequences of active receptor-like kinases are indicated below the alignments[2-6] and include: the glycine rich loop (GxGxxG), the Val-Ala-x-Lys-x-Leu (VA-K-L), glutamate (E), leucine (L), Gly-x-x-Tyr-Leu (GxxYL), His-Arg-Asp-Leu-Lys-x-x-Asn (HRDLKxxN) loop, Asp-Phe-Gly (DFG) motif,Ala-Pro-Glu (APE), Asp-x-Tyr-Ser-x-Gly (DxYSxG), andMet-x-x-x-x-x-x-x-x-x-Arg (MxxxxxxxxxR). Features and amino acids that are conserved in ARLPK1, ARLPK2, and AKIK1 are in red, while those that deviate from the consensus are boxed. Subdomains are indicated by Roman numerals.

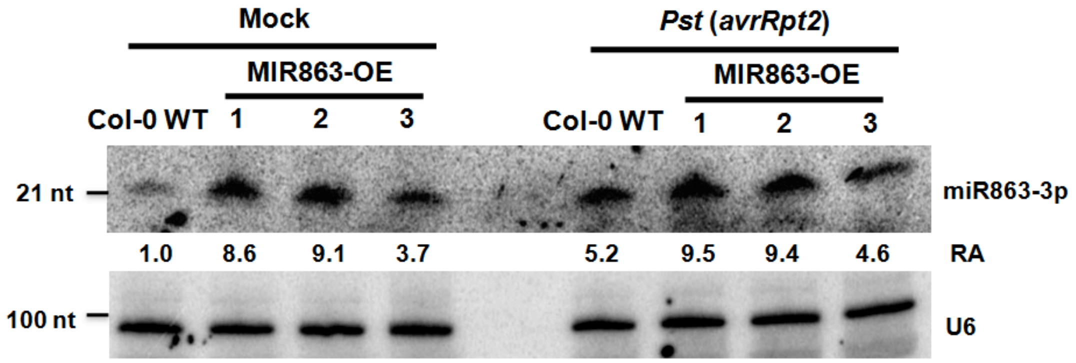

Supplementary Figure 4. Expression of miR863-3p in MIR863-OE lines after mock- or *Pst (avrRpt2)* treatment.

miR863-3p levels in mock- and *Pst* (*avrRpt2*)-infected Col-0 WT and MIR863-OE plants were detected by Northern blot. Bacterial inoculum concentration: 1 x 10^7 CFU/ml. Leaf tissue was collected at 14 hpi. U6 was used as a loading control. Relative abundance (RA) levels are indicated.

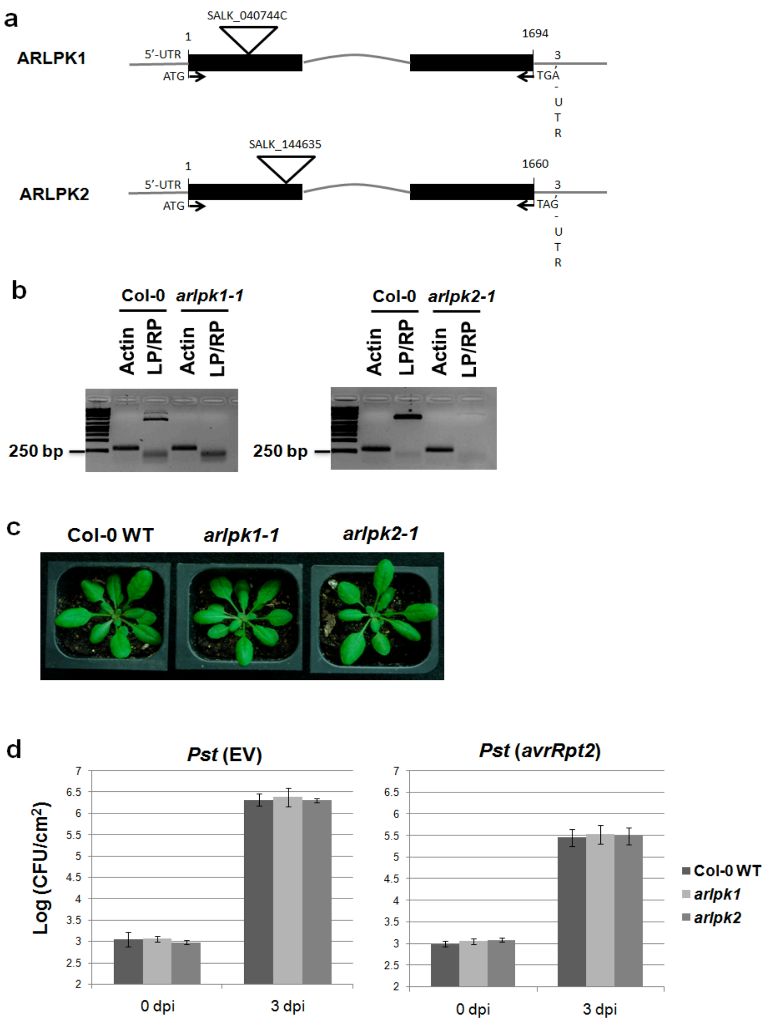

Supplementary Figure 5. Analysis *arlpk1-1*and *arlpk2-1* single mutants.

(a) Gene structure of ARLPK1 and ARLPK2 with *arlpk1-1* (SALK_040744C) and *arlpk2-1* (SALK_144635) T-DNA insertion sites indicated, respectively. Black boxes: exons, curved lines: introns, lines: UTRs, triangles: T-DNA insertion sites. Nucleotide positions are indicated by the numbers.

(b) T-DNA insertions in *arlpk1-1* (SALK_144635) and *arlpk2-1* (SALK_040744) were confirmed by real-time RT-PCR. *Actin* was used as control.

(c) Phenotypes of 4-week-old *arlpk1-1* and *arlpk2-1*mutant plants compared with Col-0 WT plants.

(d) Bacteria growth in *Pst* (EV)- and *Pst* (*avrRpt2*)-infected Col-0 WT, *arlpk1*-2, and *arlpk2-1* mutants. Bacterial inoculum concentration: 5 x 10^5 CFU/ml. Bacterial growth was measured 3 dpi. Error bars represent standard deviation for at least 15 leaf discs.

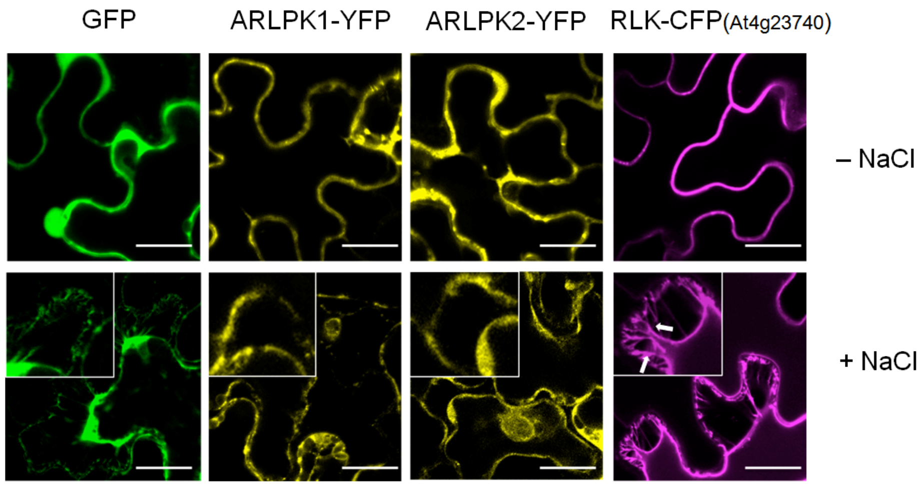

Supplementary Figure 6. ARLPK1 and ARLPK2 localize in the endoplasmic reticulum.

Subcellular localization of ARLPK1-YFP and ARLPK2-YFP in *N. benthamiana* was observed at 48 hpi with confocal microscopy. For the plasmolysis treatment, tissue was treated with 5% NaCl for 5-10 min before visualization (+ NaCl), or with water (– NaCl) as a control. GFP empty vector was used as control. RLK-CFP, a known plasma membrane (PM)-localized RLK (At4g23740) was fused to CFP at the C-terminus, was used as a PM-localization control. Arrows indicate Hechtian strands on the bottom panel. Fluorescent images were taken of the middle of the cell. Scale bars: 20 μm.

a

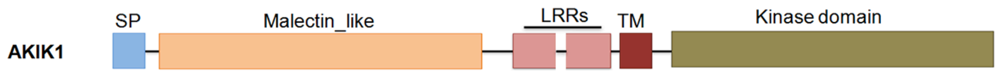

b **AKIK1**

Signal peptide

MDSPCWLLLLLLGAFAIIGCVQAQDQQEFISLDCGLPMTEPSSYTESVTGLRFSSDAEFIQTGESGKIQAS
MENDYLKPYTRLRYFPEERRNCYSLSVDKNRKYLIRARFIYGNYDGRNSNPIFELHLGPNLWATIDLQKF
VNGTMEEILHTPTSNSLNVCLVKTGTTTPLISALELRPLGNNSYLTDGSLNLFVRIYLNKKTDGFLRYPDD Malectin_like
IYDRRWHNYFMVDDWTQIFTTLEVTNDNNYEPPKKALAAAATPSNASAPLTISWPPDNPGDQYYLYSHF
SEIQDLQTNDTREFDILWDGAVVEEGFIPPKLGVTTIHNLSPVTCKGENCIYQLIKTSRSTLPSLLNALEIY
TVIQFPRNQLHLLILTSLSSTSVVAVKNIEAAYKLSRIRWQGDPCVPQKYAWDGLNCSNNTDVSKPPRVLS LRR
LNLSSSGLTGIIAAAIQNLTHLEKLDLSNNTLTGVVPEFLAQMKSLVIINLSGNNLSGPLPQGLRREGLELL
VQGNPRLCLSGSCTEKNSKKKFPVVIVASVASVAIIVAVLVIIFVLSKKKSSTVGALQPPLSMPMVHDNSP Transmembrane domain
EPSIETKKRRFTYSEVIKMTNNFQRVVGEGGFGVVCHGTINGSEQVAVKVLSQSSSQGYKHFKAEVDLL
LRVHHTNLVSLVGYCDERDHLALIYEFLPKGDLRQHLSGKSGGSFINWGNRLRIALEAALGLEYLHSGC
Kinase domain
TPPIVHRDIKTTNILLDEQLKAKLADFGLSRSFPIGGETHISTVVAGTPGYLDPEYYQTTRLGEKSDVYSF
GIVLLEIITNQPVIDQSRSKSHISQWVGFELTRGDITKIMDPNLNGDYESRSVWRVLELAMSCANPSSVNR
PNMSQVANELKECLVSENLRENMNMDSQNSLKVSMSFDTELFPRAR

Supplementary Figure 7. AKIK1 is a receptor-like kinase.

(a) Diagram of AKIK1 with important predicted domains indicated. SP (blue box): signal peptide; malectin-like (peach box); LRR (pink box): Leucine-rich repeat; TM (red box): transmembrane domain; kinase domain (brown box).

(b) Amino acid sequence of AKIK1 with signal peptide (blue), malectin-like domain (peach); LRR domains (pink), transmembrane domain (red), and kinase domains (brown) indicated.

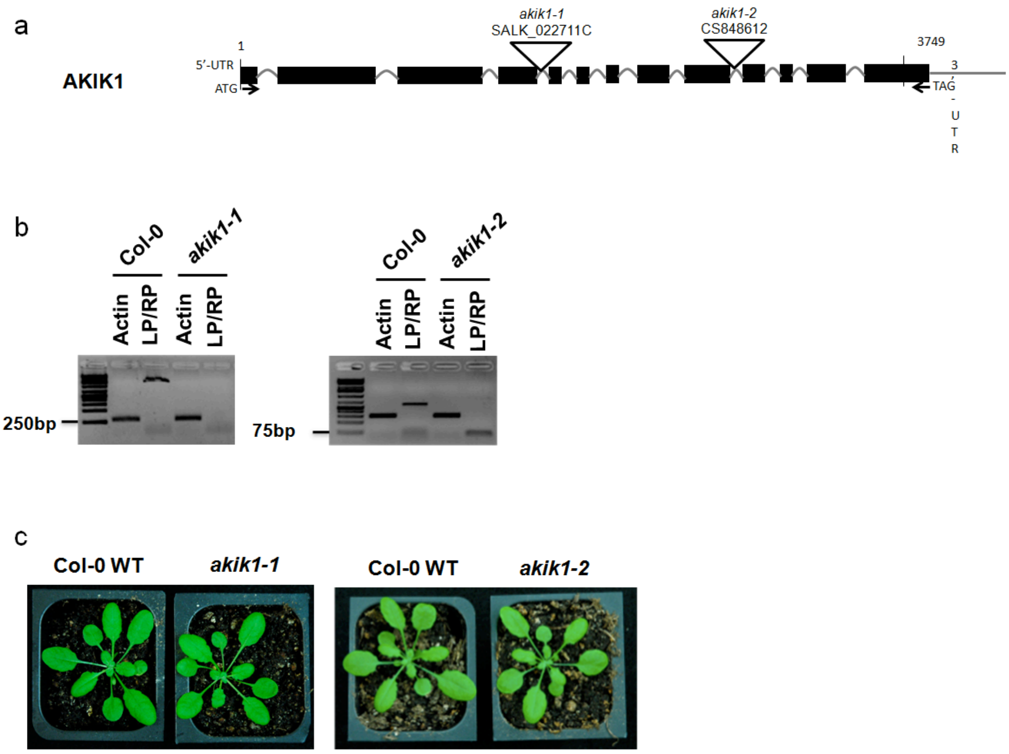

Supplementary Figure 8. Analysis of *akik1-1* and *akik1-2* mutants.

(a) Gene structure of AKIK1 with *akik1-1* (SALK_022711C) and *akik1-2* (CS848612) T-DNA insertion sites indicated. Black boxes: exons, curved lines: introns, lines: UTRs, triangles: T-DNA insertion sites. Nucleotide positions are indicated by the numbers.

(b) T-DNA insertions in *akik1-1* and *akik1-2* were confirmed by real-time RT-PCR. Actin was used as control.

(c) Phenotypes of 4-week-old *akik1-1*and *akik1-2* mutants compared with Col-0 WT plants.

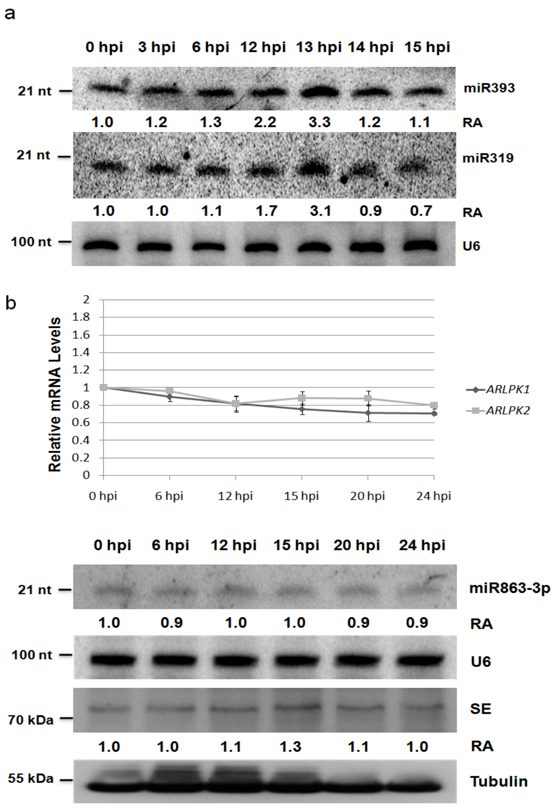

Supplementary Figure 9. Expression levels of miR393 and miR319 and *ARLPK1*, *ARLPK2*, and SE in Col-0 WT plants infected with various strains of *Pst.*

(a) Time course of miR393 and mR319 levels in *Pst* (*avrRpt2*)-infected Col-0 WT plants were detected by Northern blot analysis. U6 was used as loading control. Relative abundance (RA) levels are indicated. Bacterial inoculum concentration: 5×10^6 CFU/ml.

(b) Top: Time course of relative expression levels of *ARLPK1* and *ARLPK2* transcripts in *Pst* (EV)-infected Col-0 WT plants were detected by real-time RT-PCR. Bacterial inoculum concentration: 5×10^6 CFU/ml. Error bars indicate standard deviation from three technical replicates. Bottom: Time course of miR863-3p levels in *Pst* (EV)-infected Col-0 WT plants were detected by Northern blot. U6 was used as loading control. SE protein levels in *Pst* (EV)-inoculated Col-0 WT plants were detected by Western blot using an anti-SE antibody. α-Tubulin was used as a loading control. Relative abundance (RA) levels are indicated. Bacterial inoculum concentration: 5×10^6 CFU/ml.

Supplementary Figure 10. Uncropped images of each gel and blot presented in the manuscript with corresponding Figure numbers indicated.

For Figure 1a–c

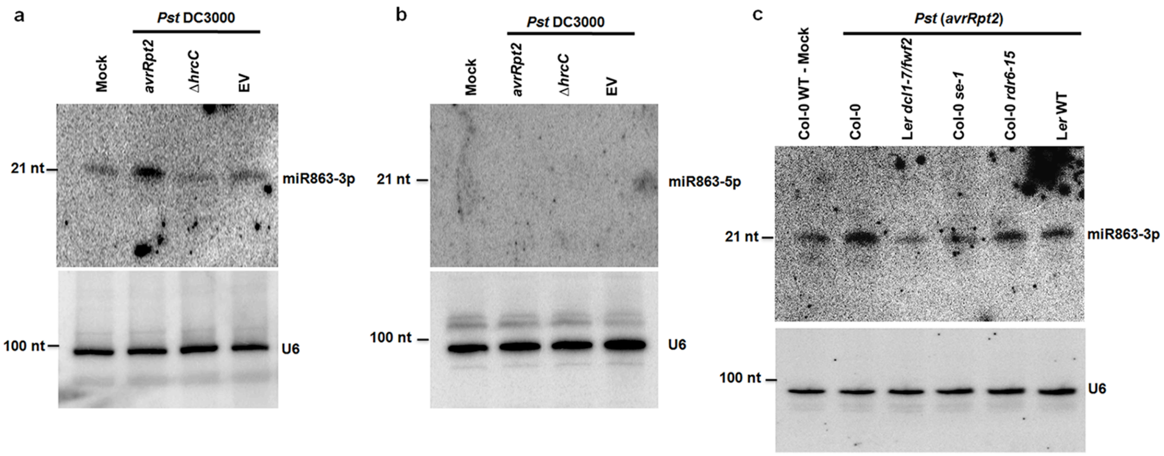

For Figure 2c,e,f

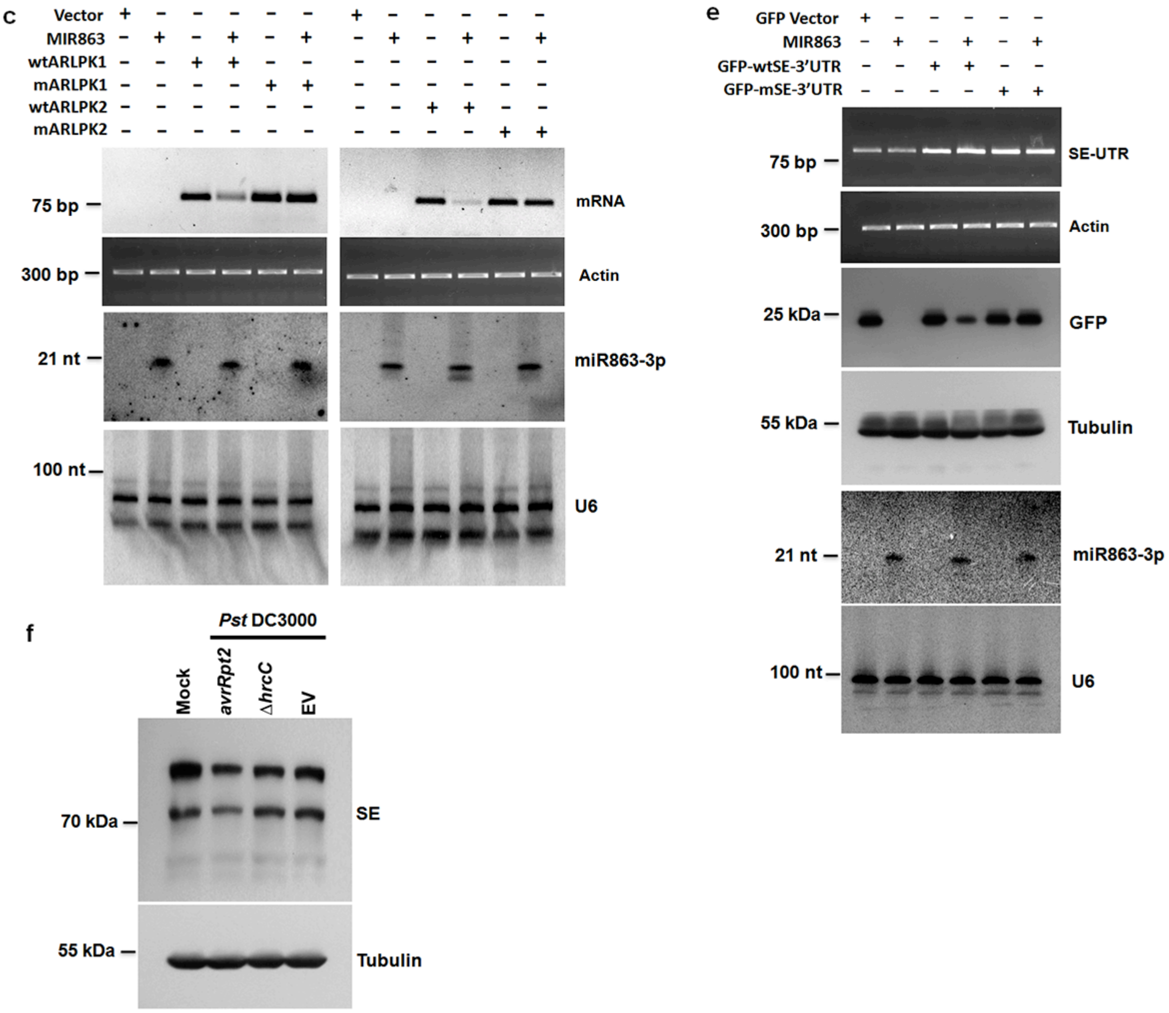

For Figure 3a–b

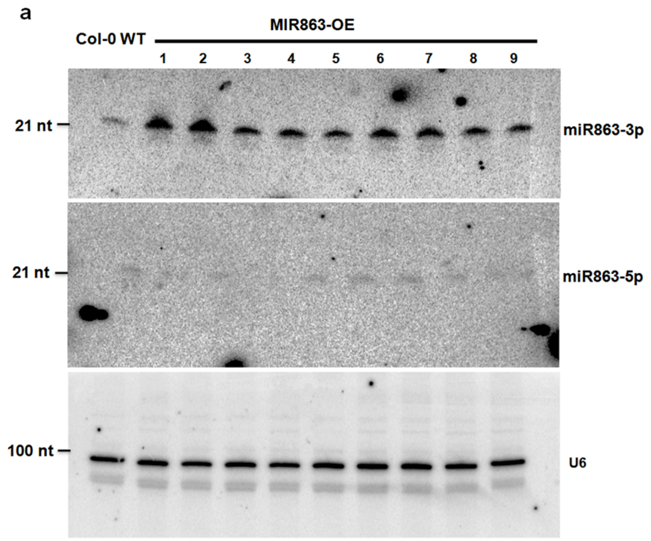

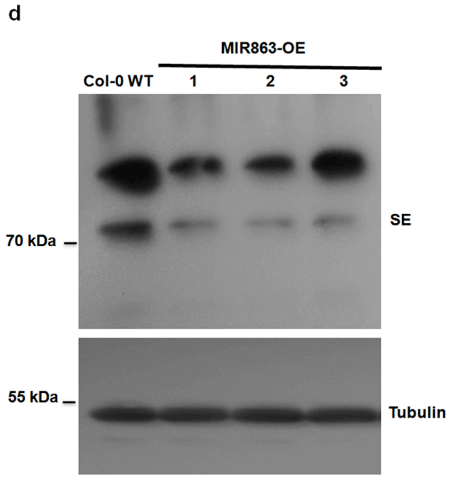

For Figure 4c,f

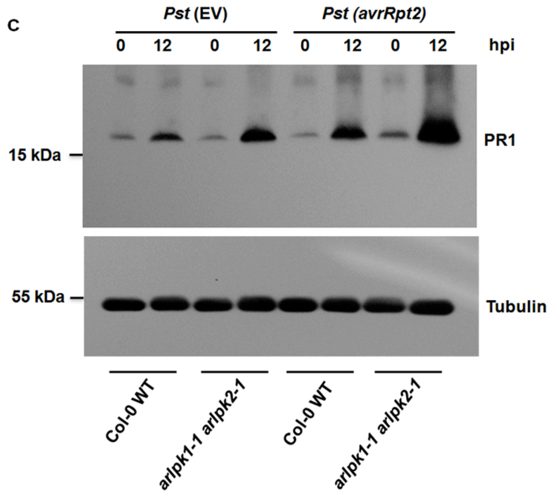

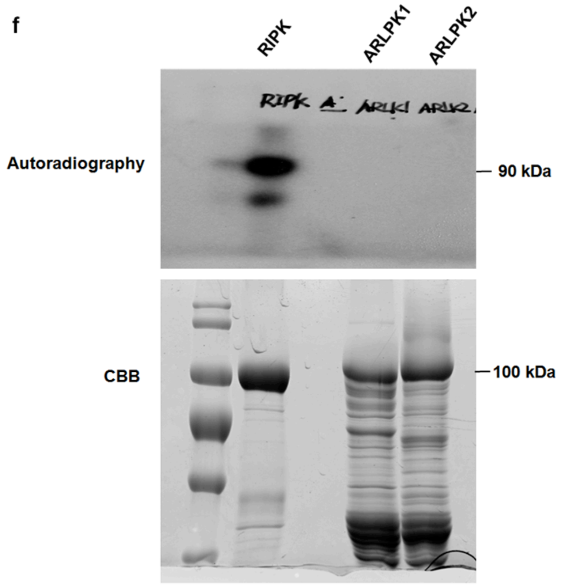

For Figure 6a,c,e,f

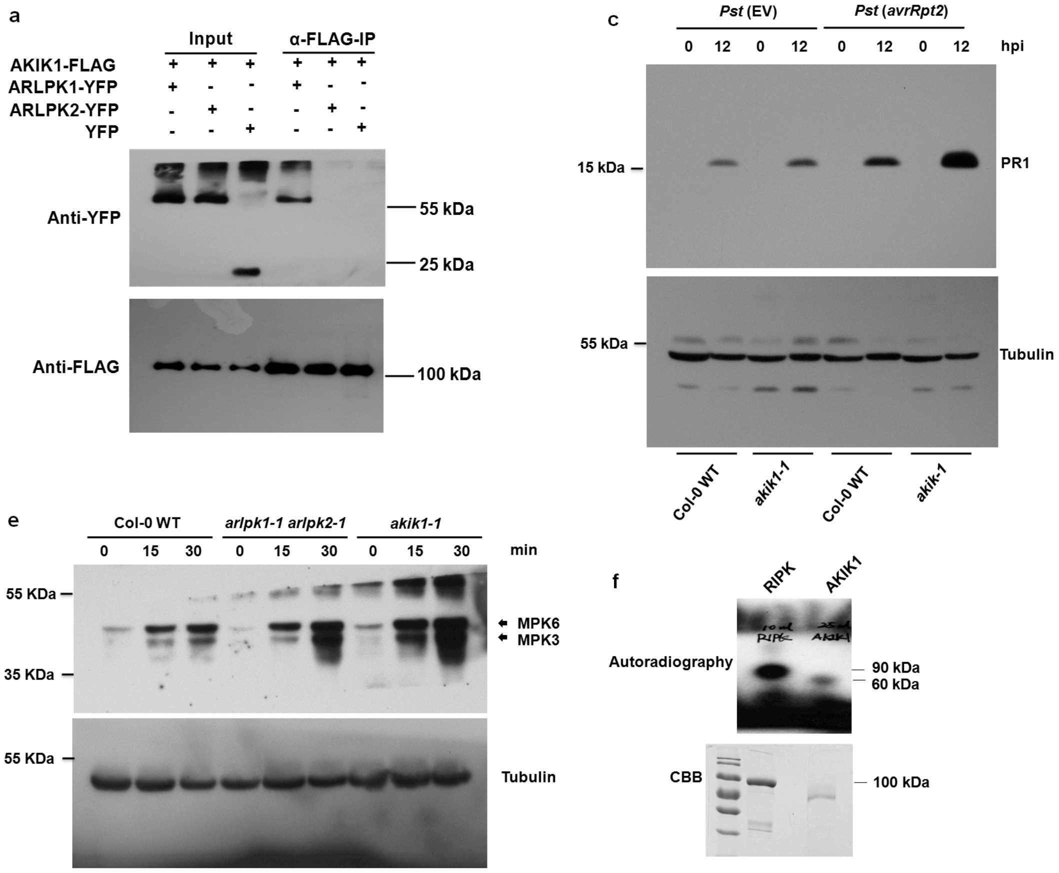

For Figure 7d,f,h

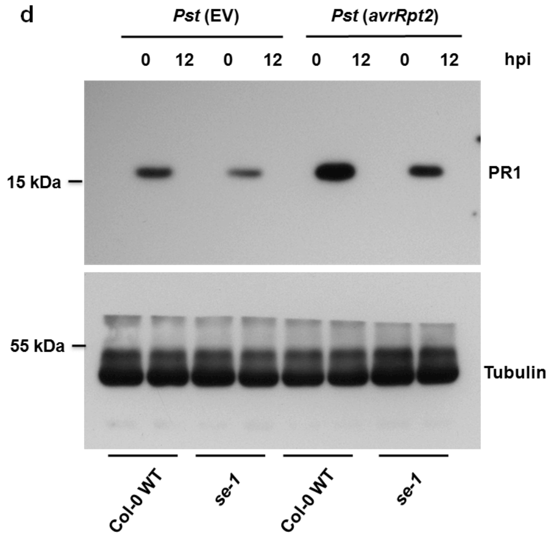

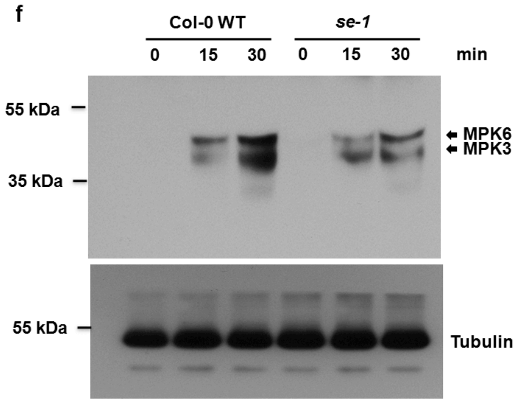

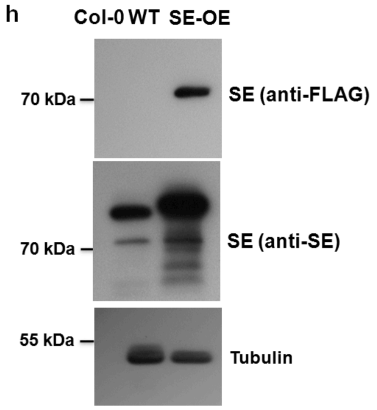

For Figure 8b,c

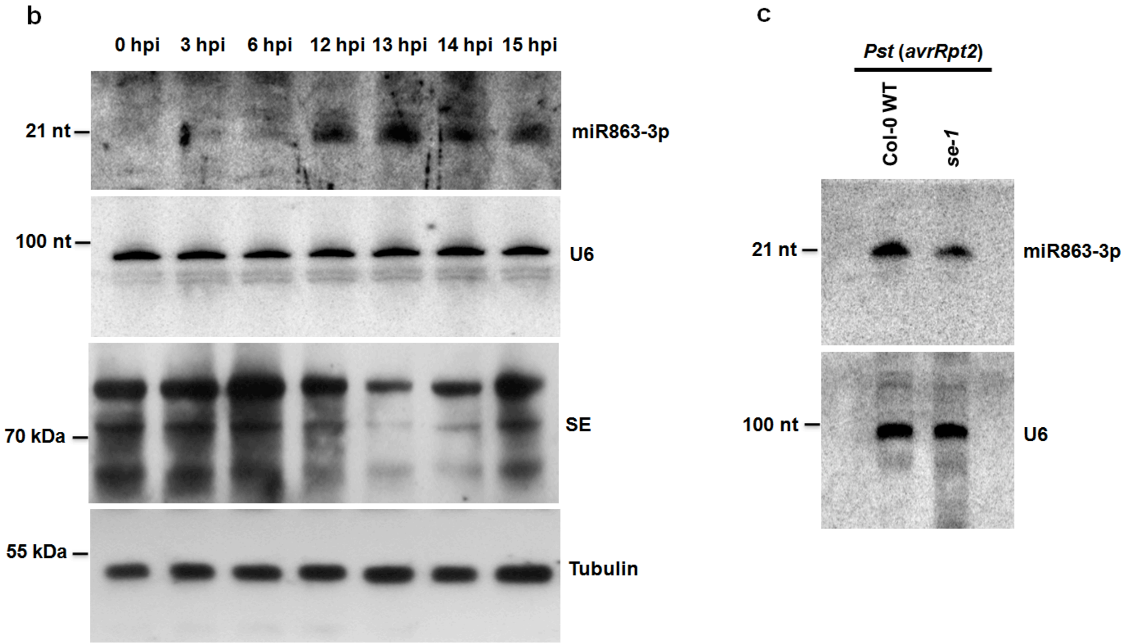

For Supplementary Figure 1b

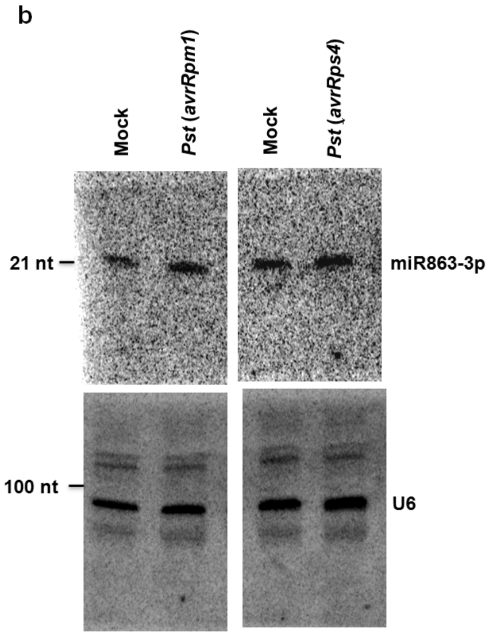

For Supplementary Figure 4

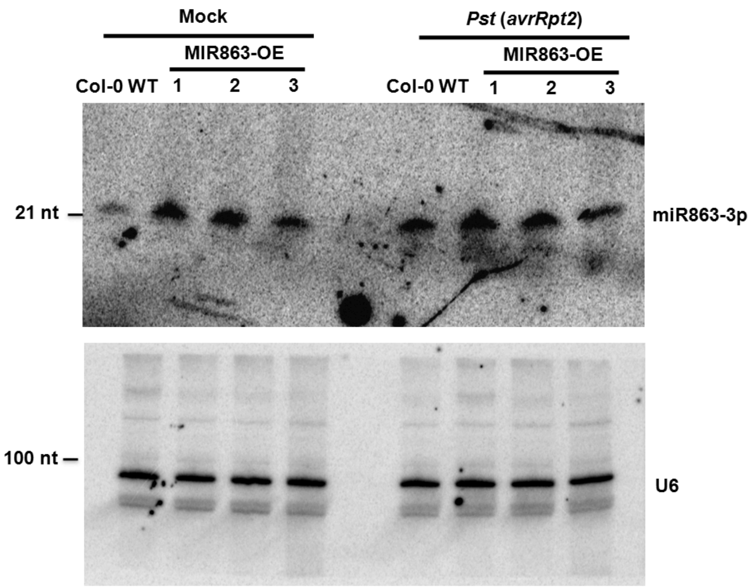

For Supplementary Figure 9a,b

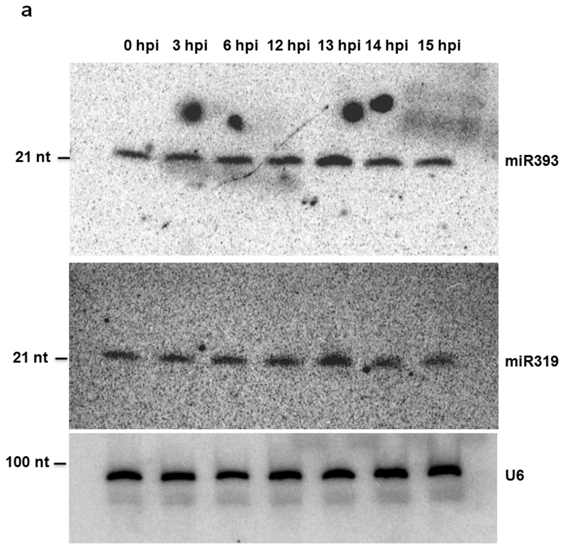

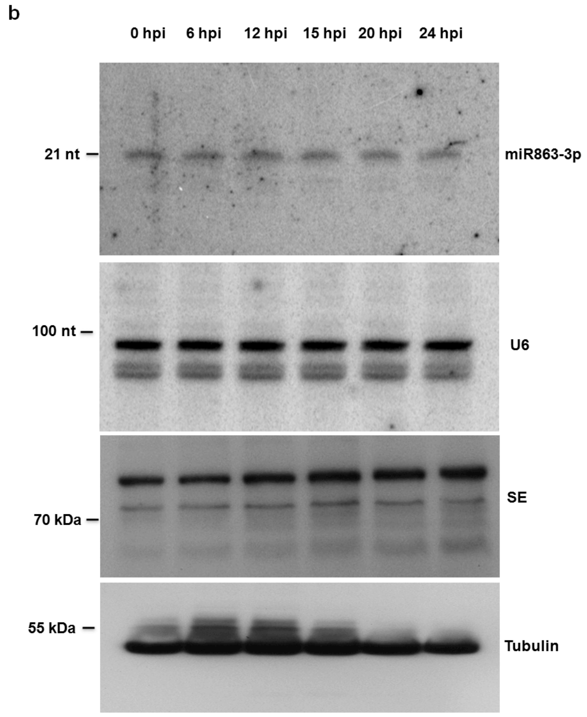

Supplementary Figure 11. Results for biological replicates conducted in this study with corresponding figure numbers indicated.

Figure 1a–c

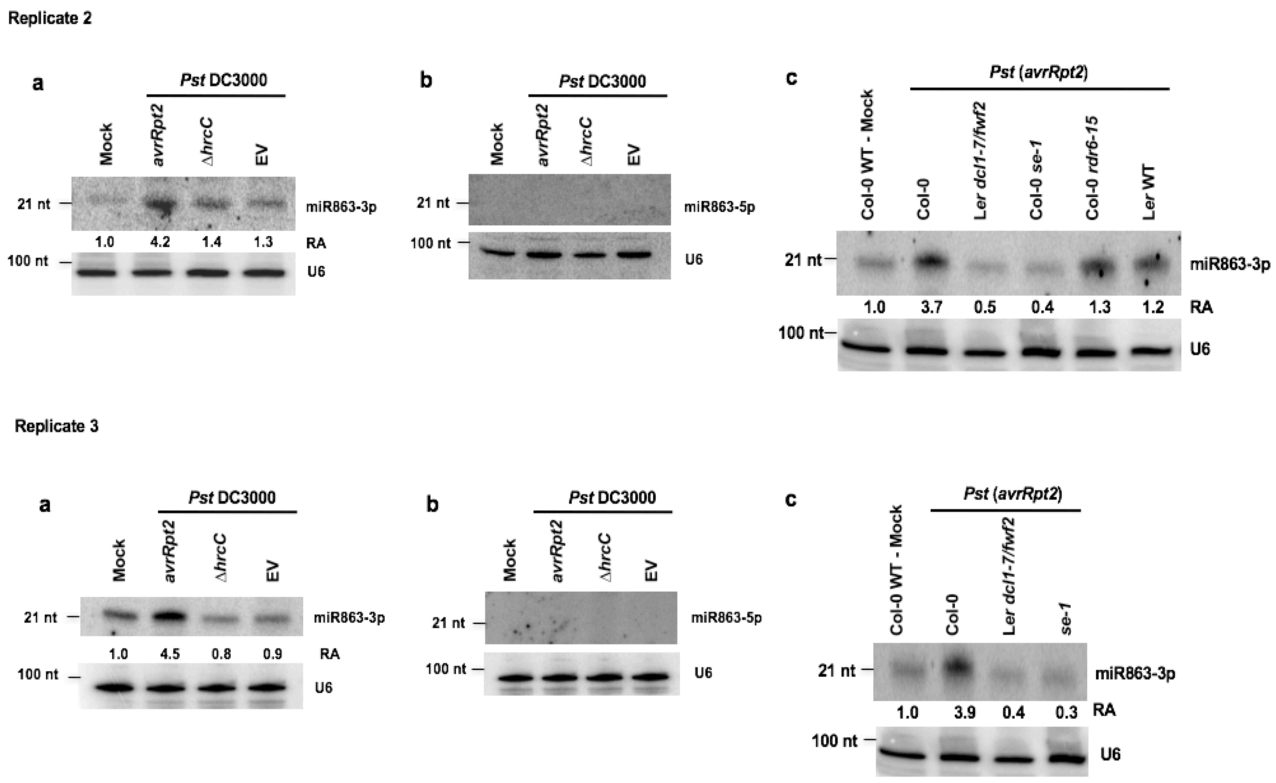

Figure 2b

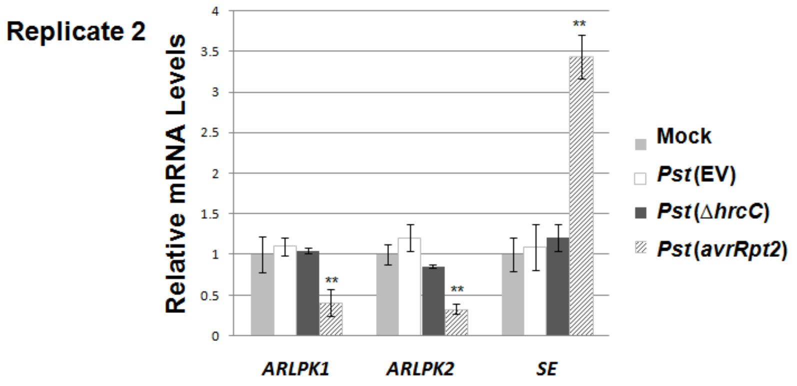
Replicate 2
Relative mRNA Levels
0
0.5
1
1.5
2
2.5
3
3.5
4
**
**
**
ARLPK1
ARLPK2
SE
Mock
Pst(EV)
Pst(ΔhrcC)
Pst(avrRpt2)

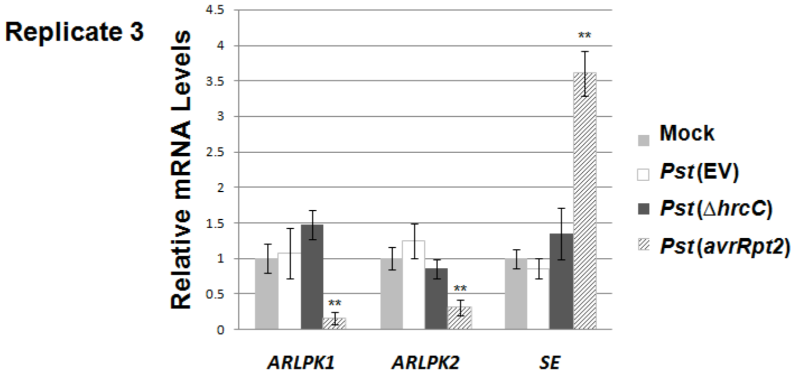
Replicate 3
Relative mRNA Levels
0
0.5
1
1.5
2
2.5
3
3.5
4
4.5
**
**
**
ARLPK1
ARLPK2
SE
Mock
Pst(EV)
Pst(ΔhrcC)
Pst(avrRpt2)

Figure 2c, 2e

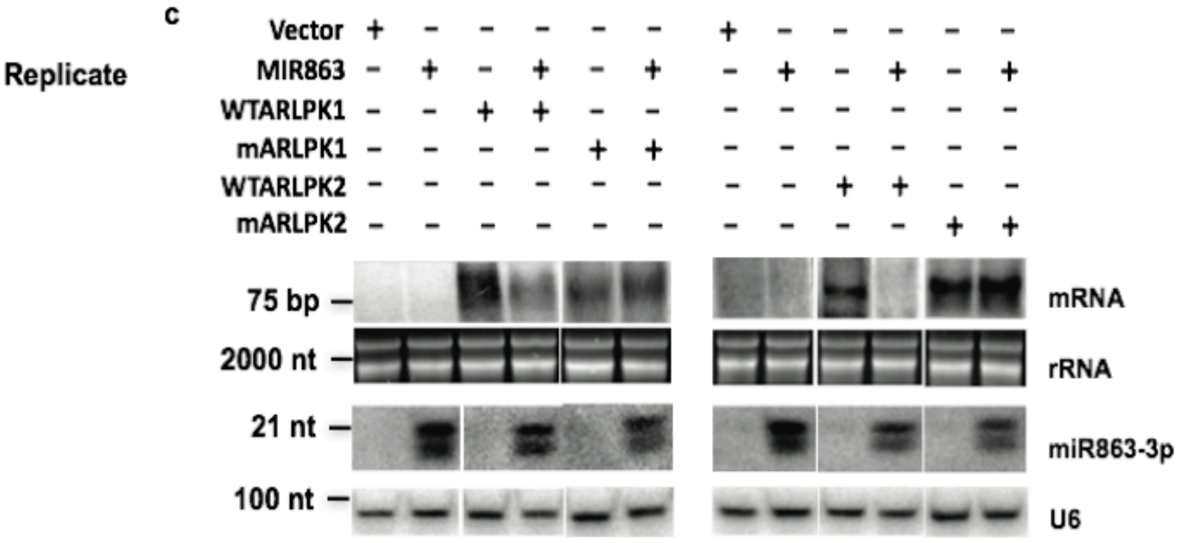
c
Replicate
Vector
MIR863
WTARLPK1
mARLPK1
WTARLPK2
mARLPK2
75 bp
2000 nt
21 nt
100 nt
mRNA
rRNA
miR863-3p
U6

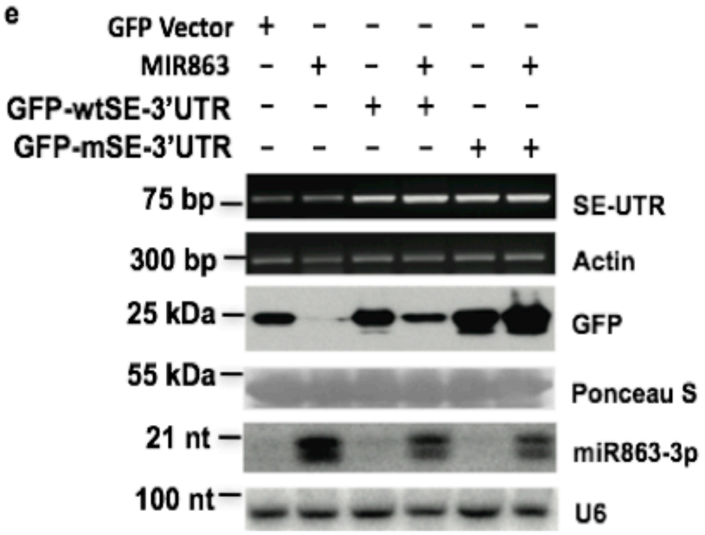
e
GFP Vector
MIR863
GFP-wtSE-3'UTR
GFP-mSE-3'UTR
75 bp
300 bp
25 kDa
55 kDa
21 nt
100 nt
SE-UTR
Actin
GFP
Ponceau S
miR863-3p
U6

Figure 2f

Replicate 2

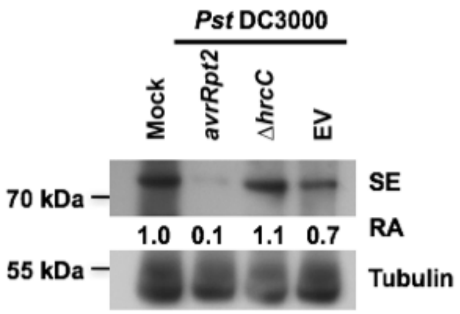

Replicate 3

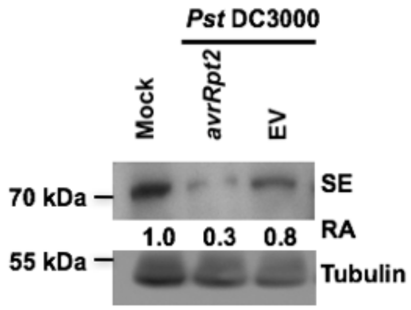

Figure 3a

Replicate

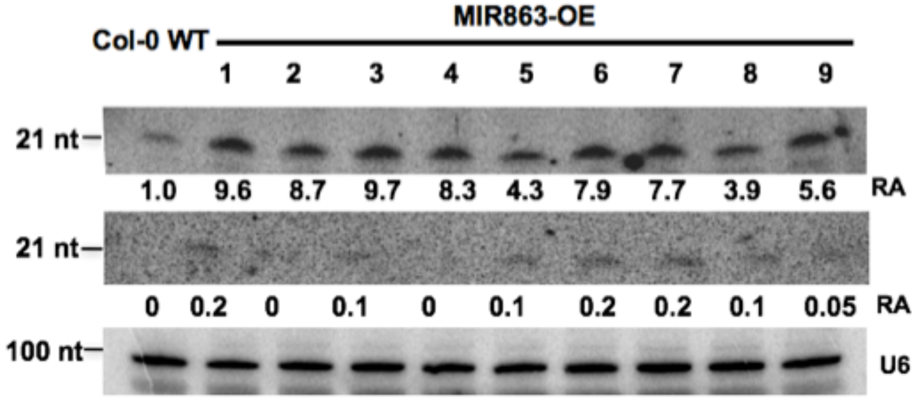

Figure 3c

Replicate 2

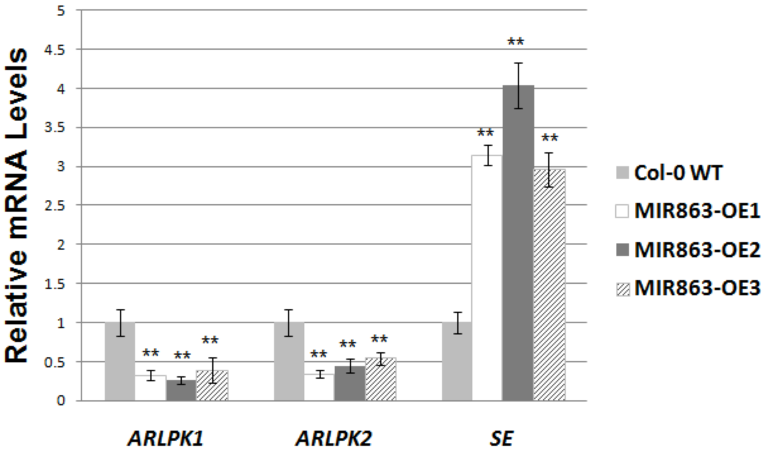

Replicate 3

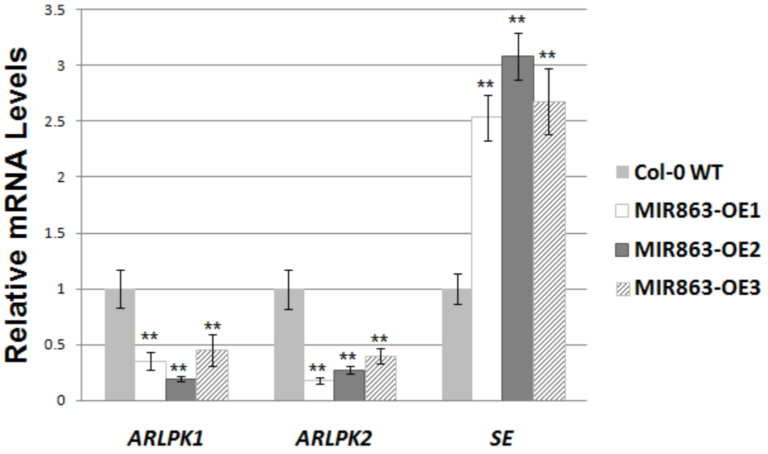

Figure 3d

Replicate

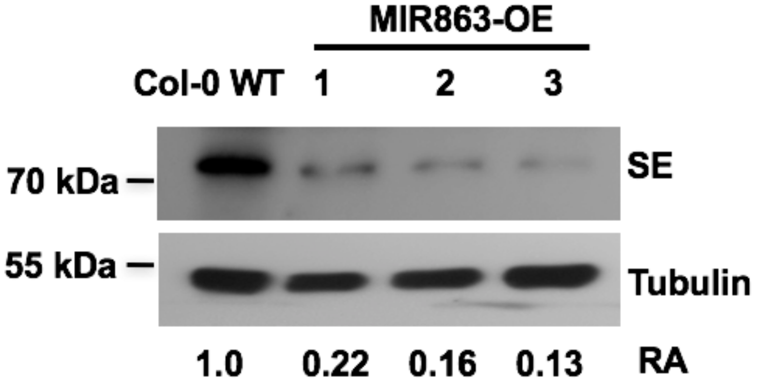

Figure 3e

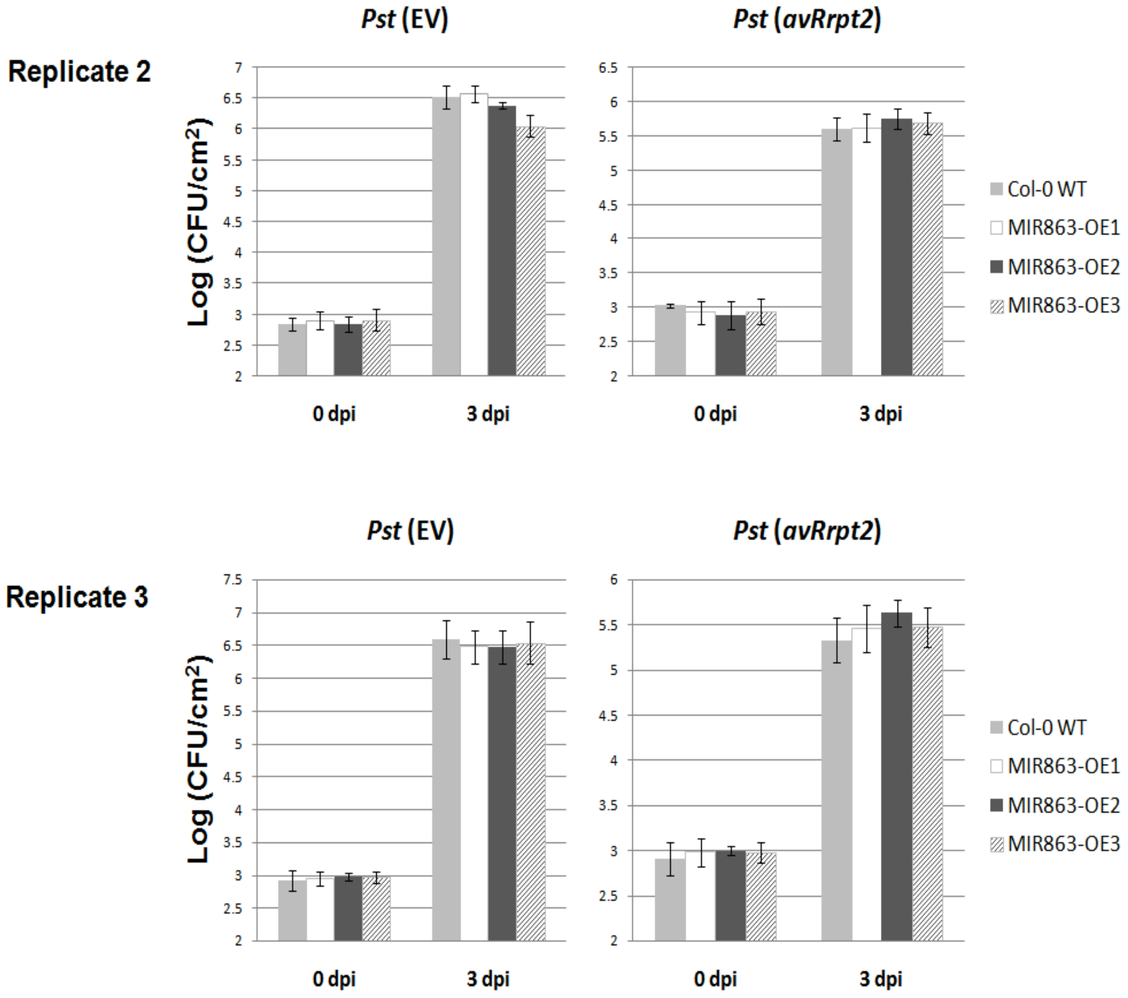

Replicate 2
Pst (EV)
Pst (avRrpt2)
Log (CFU/cm2)
0 dpi
3 dpi
Col-0 WT
MIR863-OE1
MIR863-OE2
MIR863-OE3
Replicate 3
Pst (EV)
Pst (avRrpt2)
Log (CFU/cm2)
0 dpi
3 dpi
Col-0 WT
MIR863-OE1
MIR863-OE2
MIR863-OE3

Figure 4b

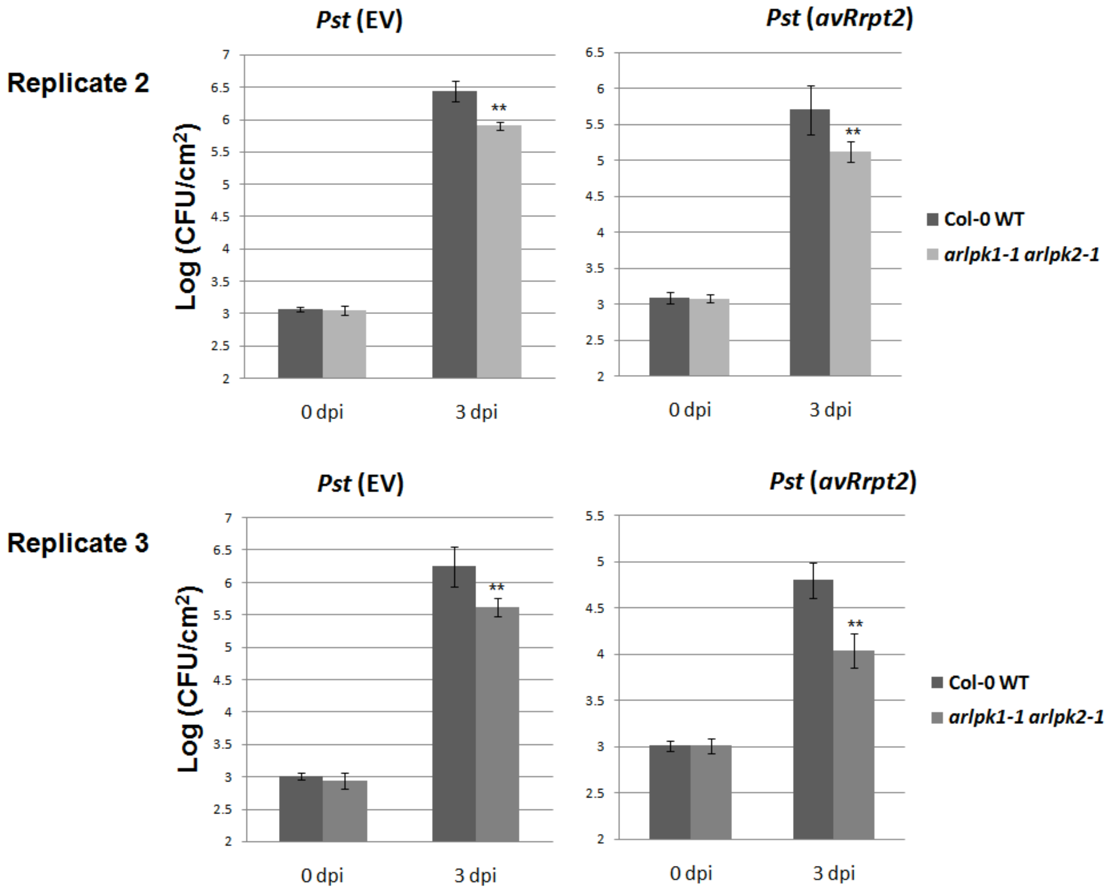
Replicate 2
Pst (EV)
Pst (avRrpt2)
Log (CFU/cm2)
0 dpi
3 dpi
**
Col-0 WT
arlpk1-1 arlpk2-1
Replicate 3
Pst (EV)
Pst (avRrpt2)
Log (CFU/cm2)
0 dpi
3 dpi
**
Col-0 WT
arlpk1-1 arlpk2-1

Figure 4c

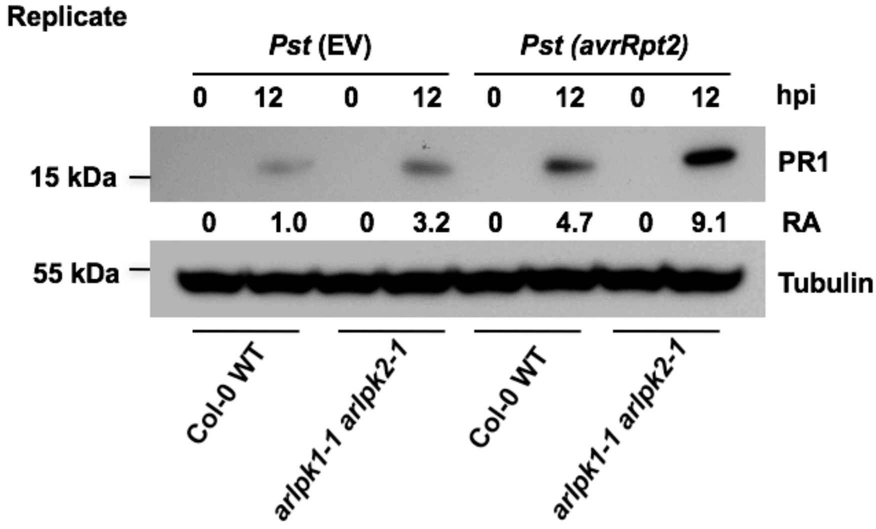

Replicate
Pst (EV)
Pst (avrRpt2)
0 12 0 12 0 12 0 12 hpi
15 kDa
PR1
0 1.0 0 3.2 0 4.7 0 9.1 RA
55 kDa
Tubulin
Col-0 WT
arlpk1-1 arlpk2-1
Col-0 WT
arlpk1-1 arlpk2-1

Figure 4d

Replicate 2

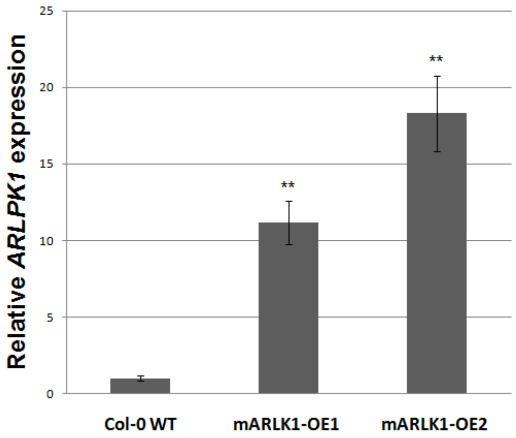

Replicate 3

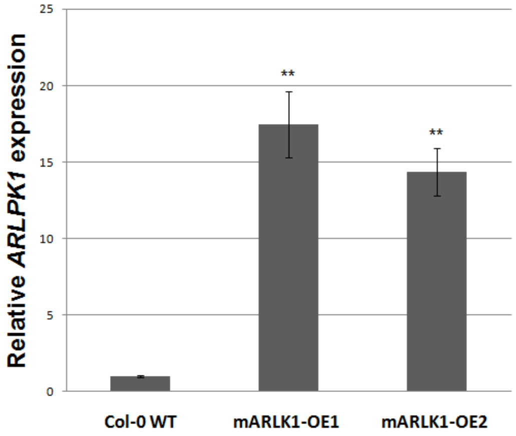

Figure 4e

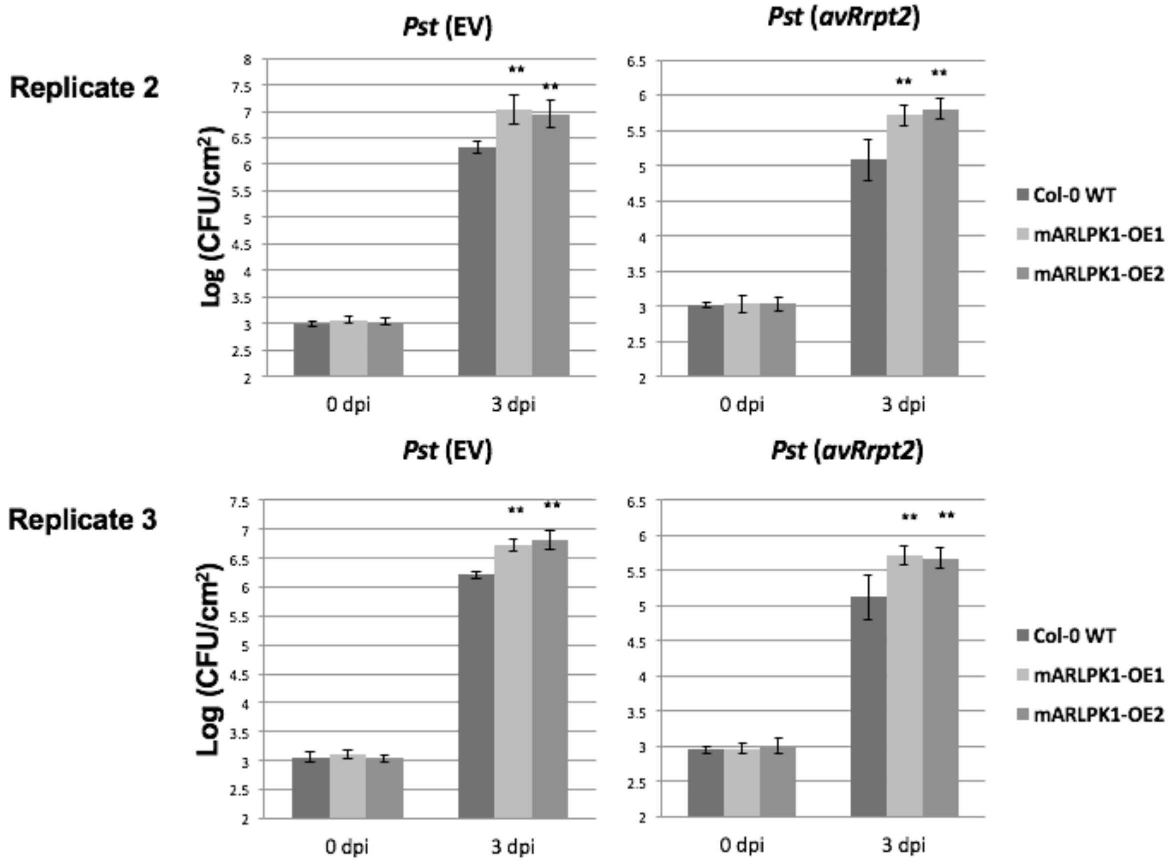
Replicate 2
Pst (EV)
Pst (avRrpt2)
Log (CFU/cm2)
0 dpi
3 dpi
**
Col-0 WT
mARLPK1-OE1
mARLPK1-OE2
Replicate 3

Figure 4f

Replicate

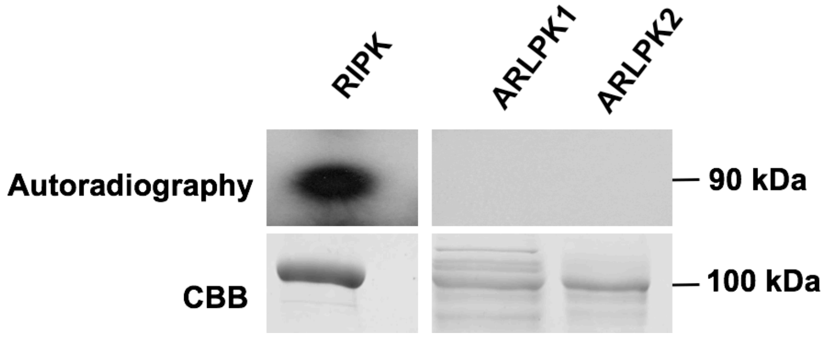

Figure 6a

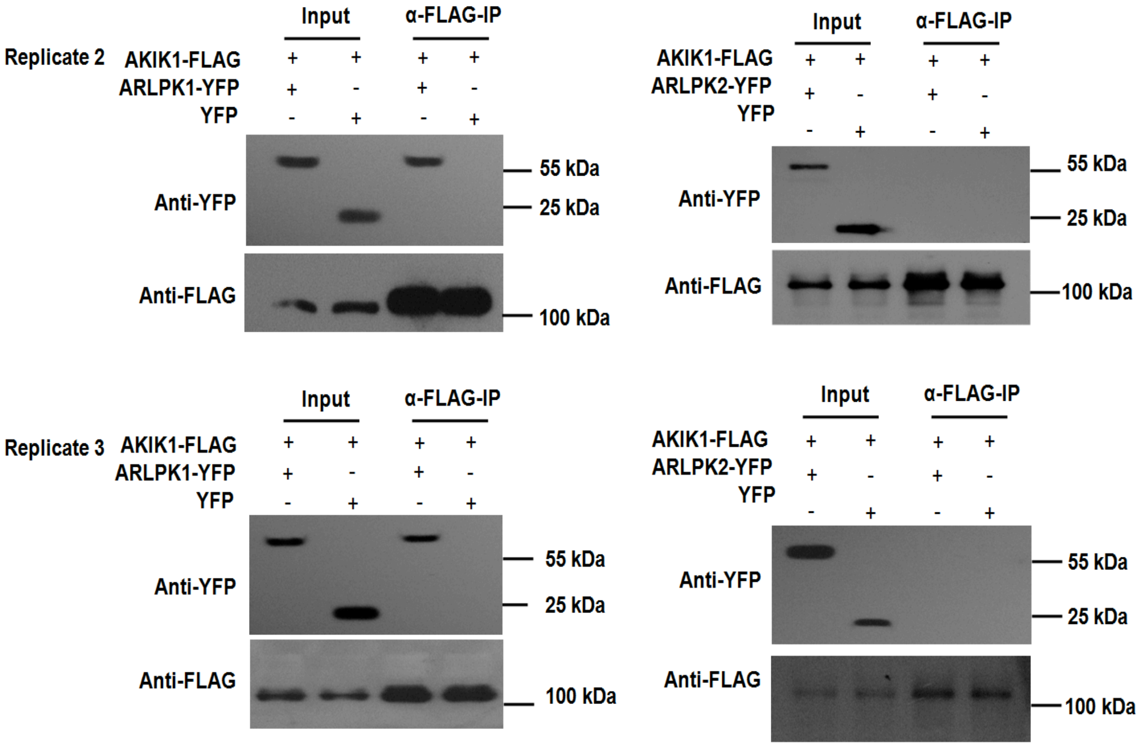
Replicate 2
Input
α-FLAG-IP
AKIK1-FLAG
ARLPK1-YFP
YFP
Anti-YFP
Anti-FLAG
55 kDa
25 kDa
100 kDa
AKIK1-FLAG
ARLPK2-YFP
YFP
Replicate 3

Figure 6b

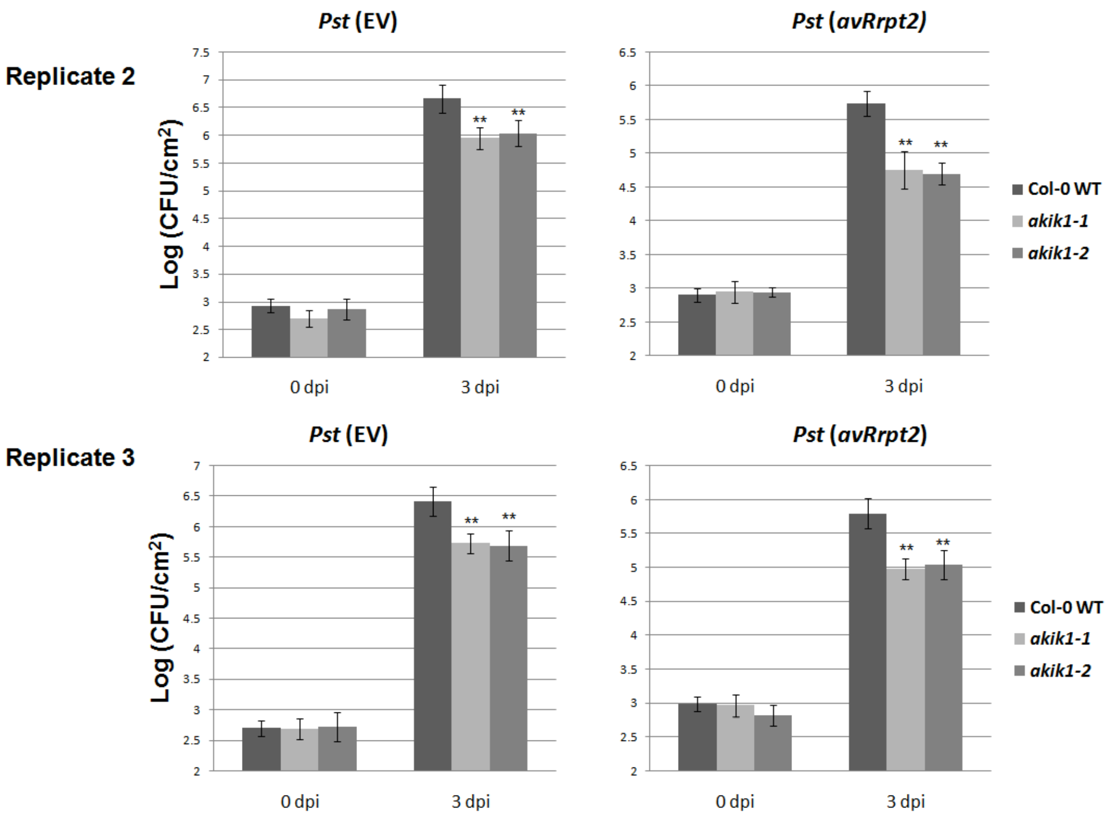
Replicate 2
Pst (EV)
Pst (avRrpt2)
Log (CFU/cm2)
0 dpi
3 dpi
Col-0 WT
akik1-1
akik1-2
Replicate 3
Pst (EV)
Pst (avRrpt2)
Log (CFU/cm2)
0 dpi
3 dpi
Col-0 WT
akik1-1
akik1-2

Figure 6c

Replicate

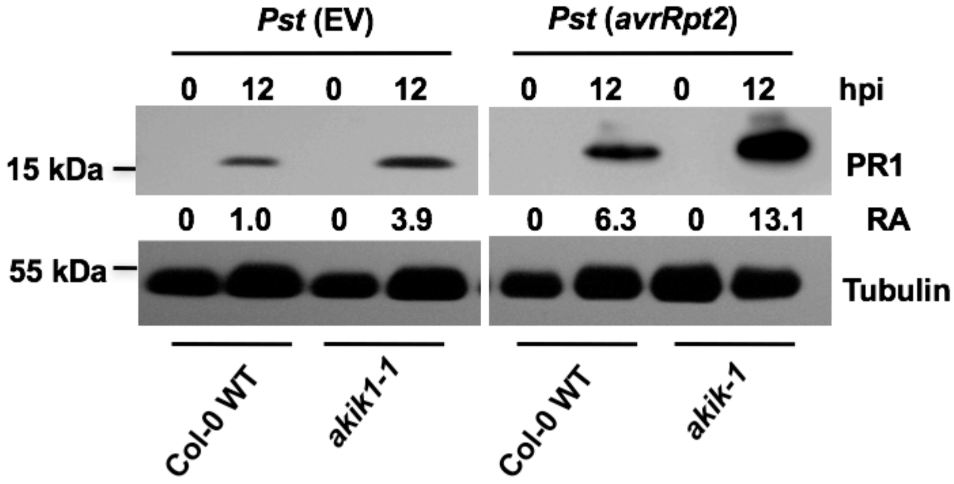

Figure 6d

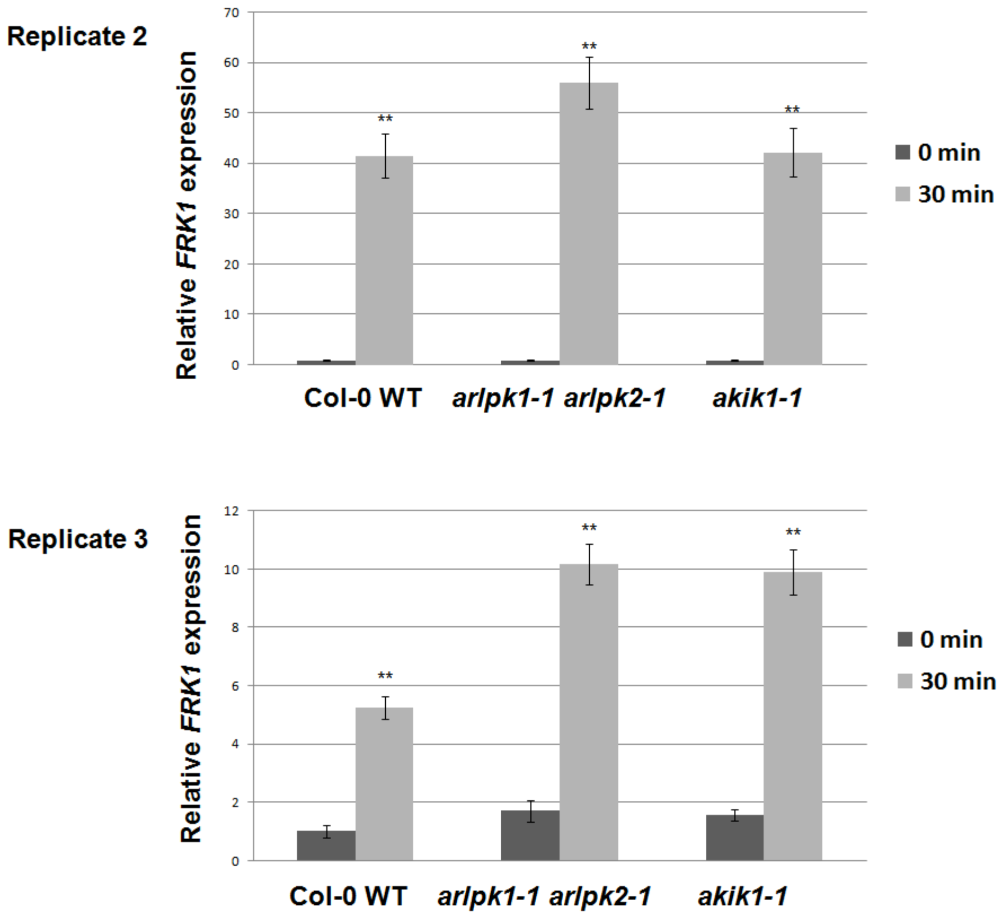
Replicate 2
Relative FRK1 expression
70
60
50
40
30
20
10
0
**
**
**
0 min
30 min
Col-0 WT
arlpk1-1 arlpk2-1
akik1-1
Replicate 3
Relative FRK1 expression
12
10
8
6
4
2
0
**
**
**
0 min
30 min
Col-0 WT
arlpk1-1 arlpk2-1
akik1-1

Figure 6e

Replicate 2

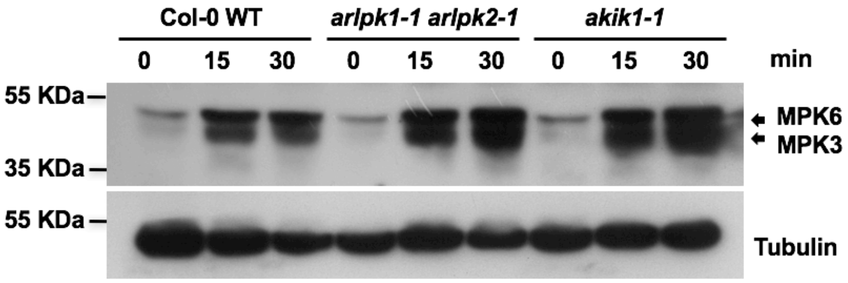

Replicate 3

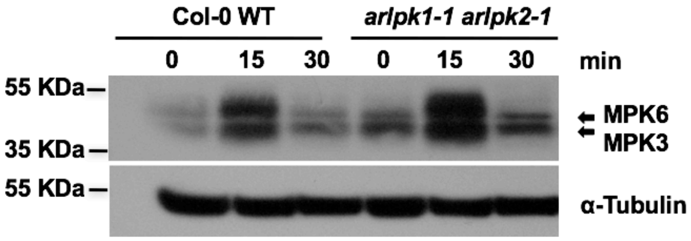

Figure 6f

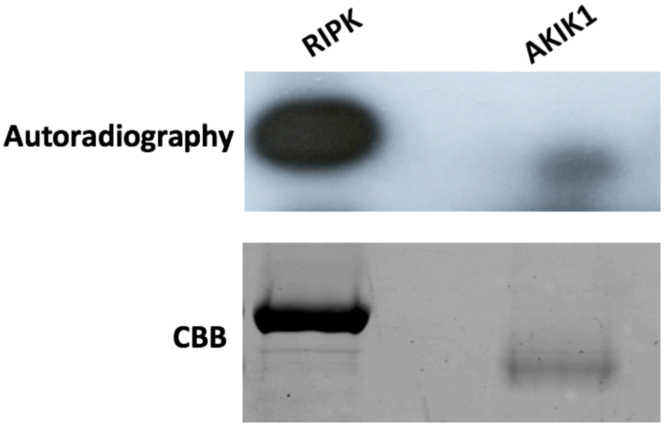

Figure 7c

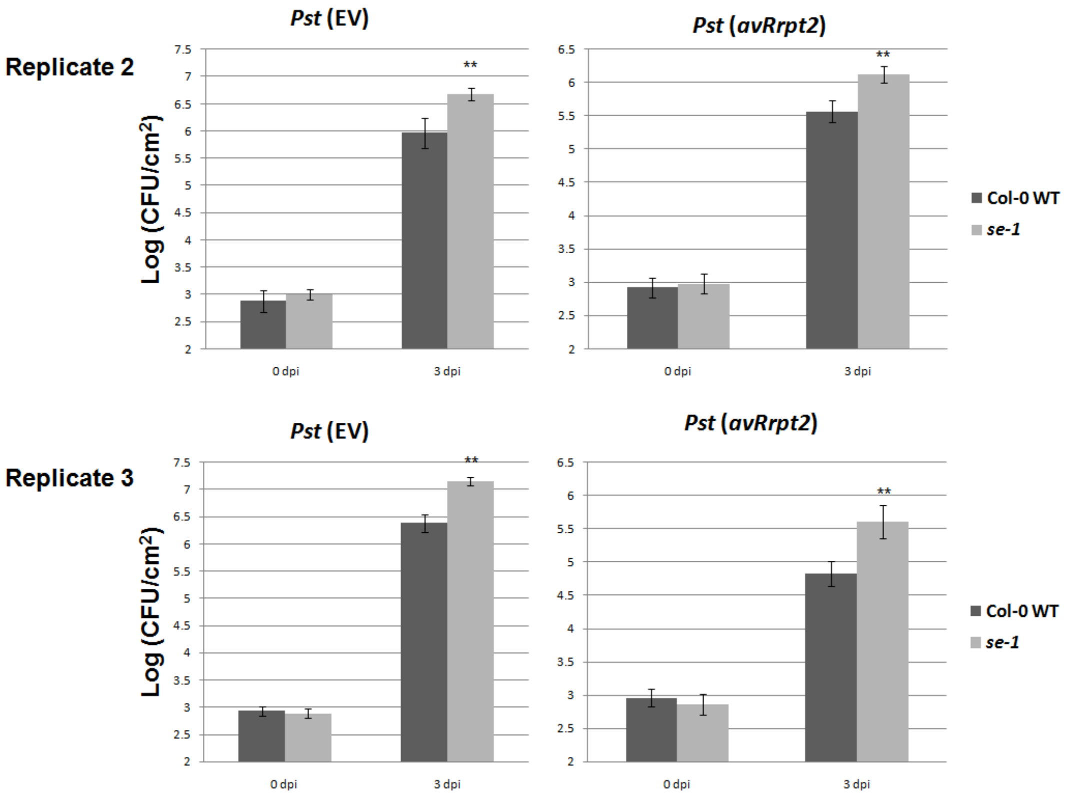
Replicate 2
Replicate 3
Pst (EV)
Pst (avRrpt2)
Log (CFU/cm2)
0 dpi
3 dpi
**
Col-0 WT
se-1

Figure 7d

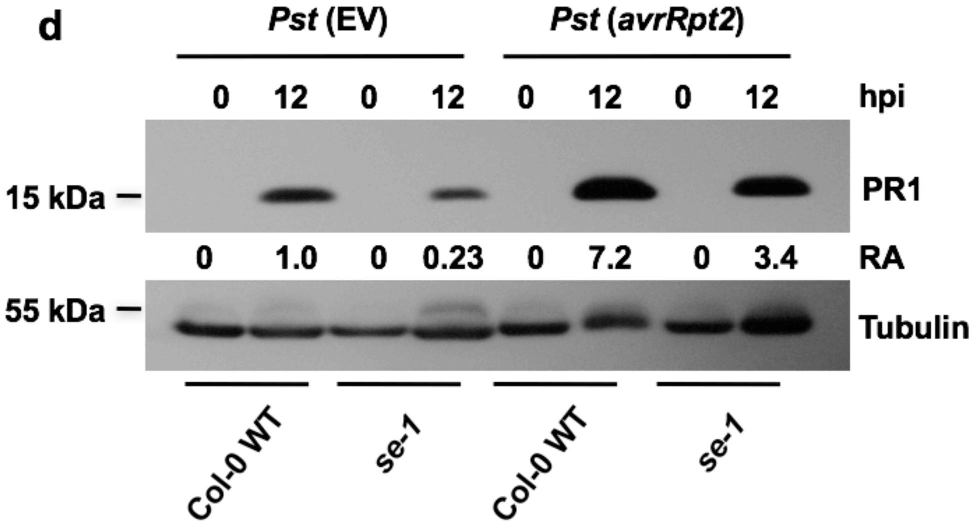

d
Pst (EV)
Pst (avrRpt2)
0 12 0 12 0 12 0 12 hpi
15 kDa
PR1
0 1.0 0 0.23 0 7.2 0 3.4 RA
55 kDa
Tubulin
Col-0 WT
se-1
Col-0 WT
se-1

Figure 7e

Replicate 2

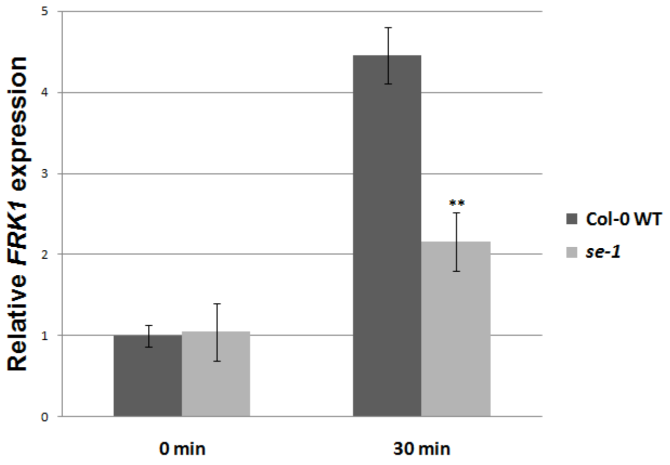

Replicate 3

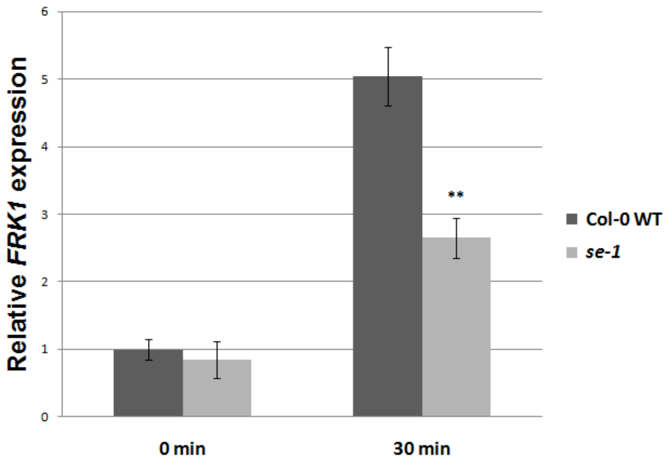

Figure 7f

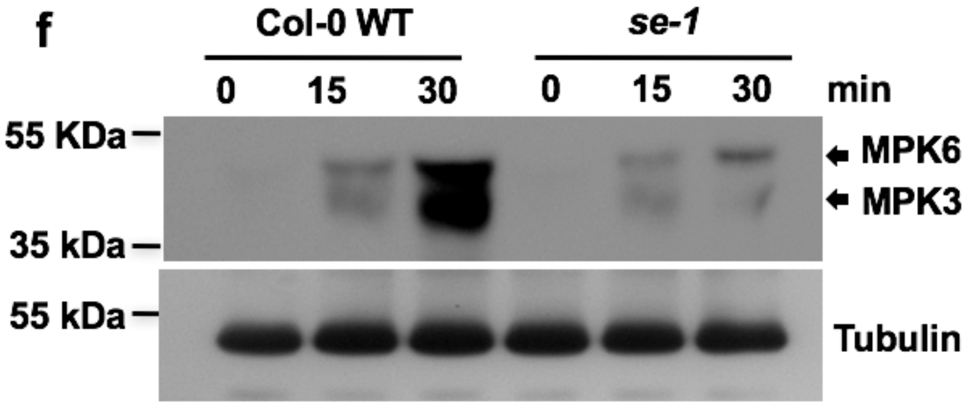

f
Col-0 WT
se-1
0
15
30
0
15
30
min
55 KDa
35 kDa
MPK6
MPK3
55 kDa
Tubulin

Figure 7h

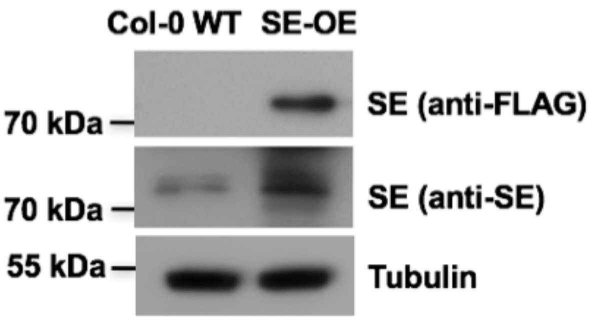

Col-0 WT
SE-OE
70 kDa
SE (anti-FLAG)
70 kDa
SE (anti-SE)
55 kDa
Tubulin

Figure 7i

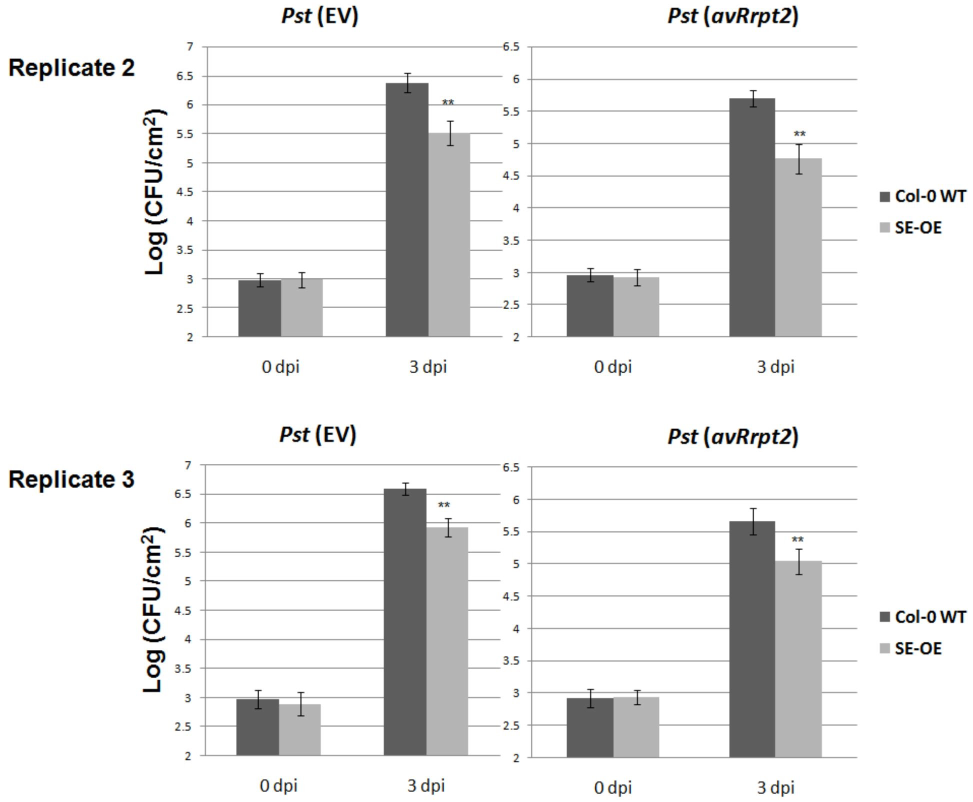
Replicate 2
Replicate 3
Pst (EV)
Pst (avRrpt2)
Log (CFU/cm2)
0 dpi
3 dpi
**
Col-0 WT
SE-OE

Figure 8a

Replicate 2

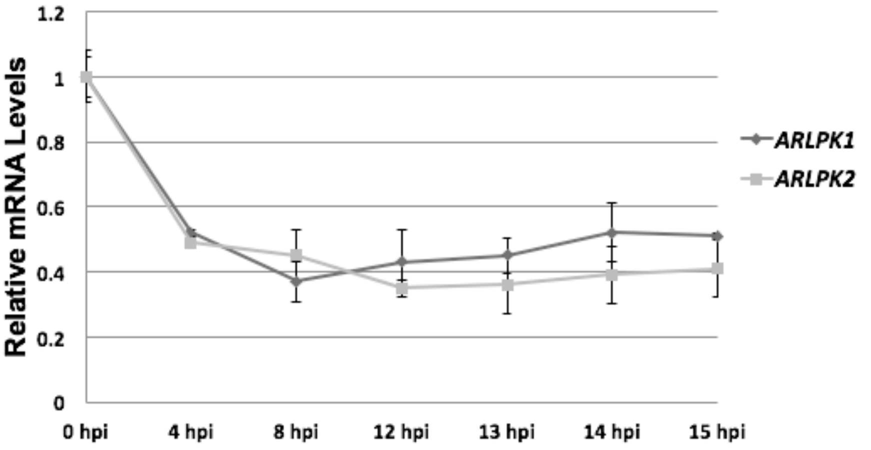

Replicate 3

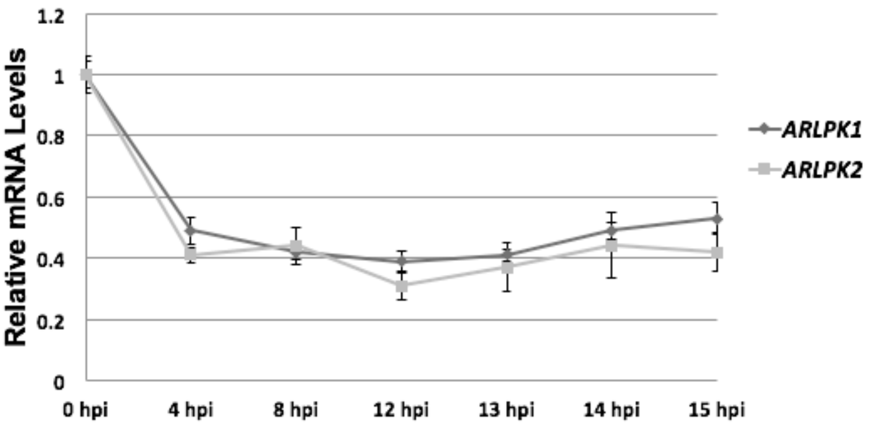

Figure 8b

Replicate

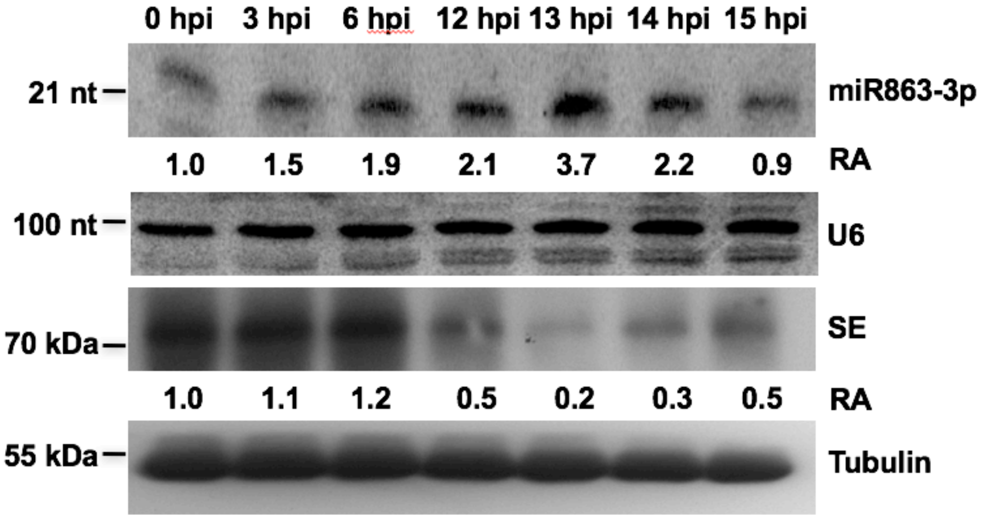

Figure 8c

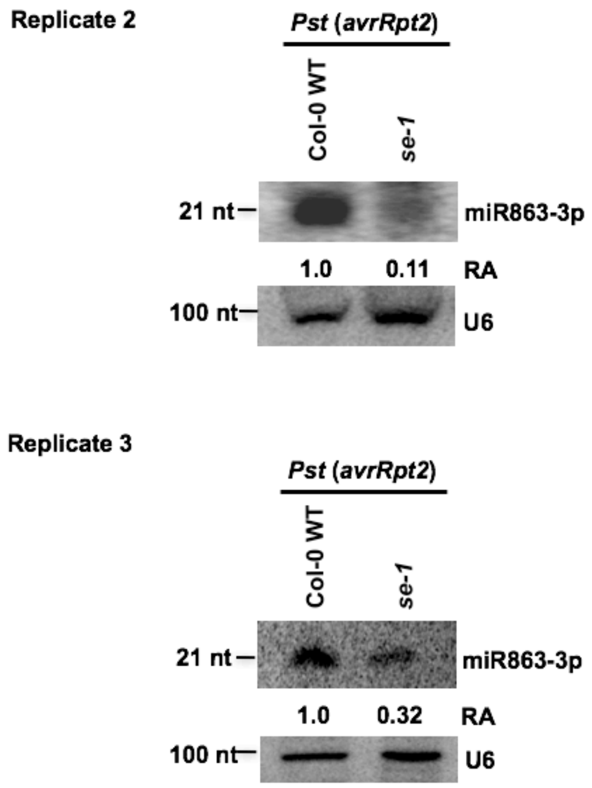
Replicate 2
Pst (avrRpt2)
Col-0 WT
se-1
21 nt
miR863-3p
1.0
0.11
RA
100 nt
U6
Replicate 3
Pst (avrRpt2)
Col-0 WT
se-1
21 nt
miR863-3p
1.0
0.32
RA
100 nt
U6

Supplementary Table 1. Primers used in this study.

Primer Name	Sequence
mARLPK1-F	5’-CTTTCAGCTTGCAATGAGCTGCTGTAGCCCTTCGCCTACTCTTAGG-3'
mARLPK1-R	5’-GAG TAGGCGAAGGGCTACAGCAGCTCATTGCAAGCTGAAAGTACTCG-3'
mARLPK2-F	5'-TTTTCAGCTTGCAATGAGCTGCTGTAGCCCTTCGCCTTCTCTTAGG-3'
mARLPK2-R	5'-GAGAAGGCGAAGGGCTACAGCAGCTCATTGCAAGCTGAAAATACTT-3'
mSE-3’UTR-F	5'-CTTTTGATGTCTGCGGGACTTCCCATACACCTTTTCACTCACTAA-3'
mSE-3’UTR-R	5'-AAGTATTGATAACAAACAATGTGGATAAAACGCATTATTCAATACAAG-3'
ARLPK1-R 5'RACE	5' TCATTTCCTGATCTCTTCAAGCTTCC 3'
ARLPK2-R 5'RACE	5' CTAAAATTTCCCAATCTCTTCAAG 3'
ACTIN2-F	5'-AGTGGTCGTACAACCGGTATTGT-3'
ACTIN2-R	5'-CTTGCCCATCGGGTAATTCATAG-3'
ARLPK1_OE-F	5'-CACCATGAGAAAATCCCATTTGGTAAA-3'
ARLPK1_OE-R	5'- TCATTTCCTGATCTCTTCAAGCTTC-3'
ARLPK2-OE-F	5'-CACCATGAGAAAATCTCTACTTCTAAC-3'
ARLPK2_OE-R	5'-CTAAAATTTCCCAATCTCTTCAAGTTT-3'
ARLPK1-qPCR-F	`5'-TTATTAGGAGATGTCTGAAGGAT-3'
ARLPK1-qPCR-R	5'-TCATTTCCTGATCTCTTCAAGCT-3'
ARLPK2-qPCR-F	5'-TGAGGAATGCTGTTCTTGATC-3'
ARLPK2-qPCR-R	5'-CTAAAATTTCCCAATCTCTTCAA-3'
AT5G59650-OE-F	5'-CACCATGGATAGTCCTTGTTGGCTTTTGCTG-3'
AT5G59650-OE-R	5'-CTACCTTGCCCTCGGAAACAA-3'
FRK1-F	5'-TGCACTTACCCTCCTTCG-3'
FRK1-R	5'-GACAGTAGAAGCCGGTTGGT-3'
LBb1.3	5'-ATTTTGCCGATTTCGGAAC-3'
SALK_022711-LP	5'-TTTGTTCCACATAGGTACCCG-3'
SALK_022711-RP	5'-GGAAGGGGACCACTAAGATTG-3'
CS848612-LP	5'-ACCTCCACTGAGTATGCCAATG-3'
CS848612-RP	5'-ATCCAGGAGTACCAGCAACCAC-3'
MIR863-3P-OE-F	5'-CACCAGAACCCGATGGAGACCAACA-3'
MIR863-3P-OE-R	5'-CAAACCCGTCAGCTTCCAGA-3'
MIR863-3P-probe	5'-ATTATGTCTTGTTGCTCTCAA-3'
MIR863-5P-probe	5'-TATTGAGATCAACAAGACATA-3'
SALK_144635-LP	5'-CCCTTACAAGGCCAAGAAATC-3'
SALK_144635-RP	5'-AGCTGTGTGGTTCTCTTTTGG-3'

SALK_040744C-LP	5'-TTTTGGGAGGTGATTTCGTAC-3'
SALK_040744C-RP	5'-AATCTTTTTCAGCTTCCTCGC-3'
SERRATE-qPCR-F	5'-ATGATCCTAATGCTCCAGGAG-3'
SERRATE-qPCR-R	5'-CGTCTAGGATCTTGTCTAAAG-3'

Supplementary References

1. Sievers, F. *et al.* Fast, scalable generation of high-quality protein multiple sequence alignments using Clustal Omega. *Mol Syst Biol* **7**, 539 (2011).
2. Kornev, A.P. & Taylor, S.S. Defining the conserved internal architecture of a protein kinase. *Biochim. Biophys. Acta* **1804**, 440-4 (2010).
3. Endicott, J.A., Noble, M.E. & Johnson, L.N. The structural basis for control of eukaryotic protein kinases. *Annu. Rev. Biochem.* **81**, 587-613 (2012).
4. Hanks, S.K., Quinn, A.M. & Hunter, T. The protein kinase family: conserved features and deduced phylogeny of the catalytic domains. *Science* **241**, 42-52 (1988).
5. Hanks, S.K. & Hunter, T. Protein kinases 6. The eukaryotic protein kinase superfamily: kinase (catalytic) domain structure and classification. *FASEB J.* **9**, 576-96 (1995).
6. Boudeau, J., Miranda-Saavedra, D., Barton, G.J. & Alessi, D.R. Emerging roles of pseudokinases. *Trends Cell Biol.* **16**, 443-52 (2006).
